# Morphogen gradients are regulated by porous media characteristics of the developing tissue

**DOI:** 10.1242/dev.204312

**Published:** 2025-07-14

**Authors:** Justina Stark, Rohit Krishnan Harish, Ivo F. Sbalzarini, Michael Brand

**Affiliations:** ^1^ Dresden University of Technology, Faculty of Computer Science, Dresden 01187, Germany; ^2^ Max Planck Institute of Molecular Cell Biology and Genetics, Dresden 01307, Germany; ^3^ Center for Systems Biology Dresden, Dresden 01307, Germany; ^4^ Center for Regenerative Therapies Dresden, Dresden 01307, Germany; ^5^ Technische Universität Dresden, Center for Molecular and Cellular Bioengineering, Dresden 01307, Germany; ^6^ Cluster of Excellence Physics of Life, TU Dresden, Dresden 01307, Germany

**Keywords:** Morphogen gradient, Extracellular space, Complex geometry, Image-based model, Reaction-diffusion, Gradient robustness, Zebrafish, Epiboly, Fibroblast growth factor, GPU-accelerated simulation

## Abstract

Long-range morphogen gradients have been proposed to form by morphogen diffusion from a localized source to distributed sinks in the target tissue. The role of the complex tissue geometry in this process is, however, less well understood and has not been explicitly resolved in existing models. Here, we numerically reconstruct pore-scale 3D geometries of zebrafish epiboly from light-sheet microscopy volumes. In these high-resolution 3D geometries, we simulate Fgf8a gradient formation in the tortuous extracellular space. Our simulations show that when realistic embryo geometries are considered, a source-diffusion-degradation mechanism with additional binding to extracellular matrix polymers is sufficient to explain emergence and robust maintenance of Fgf8a gradients. The predicted normalized gradient is robust against changes in source and sink rates but sensitive to changes in the pore connectivity of the extracellular space, with lower connectivity leading to steeper and shorter gradients. This demonstrates the importance of considering realistic geometries when studying morphogen gradients.

## Teaser

**Highlighted Article:** Image-based 3D modeling of pore-scale extracellular space geometries of the developing zebrafish embryo reveals that the Fgf8a morphogen gradient is regulated by the porous media characteristics of the developing tissue.

## INTRODUCTION

The global body plan governing cell differentiation and tissue morphogenesis during embryonic development is defined by graded concentration fields of morphogens (Wolpert, 1969; Driever and Nüsslein-Volhard, 1988; Green and Sharpe, 2015). Morphogens are signaling molecules, mostly proteins, secreted by localized source cells and moving through the embryo toward their target tissue, where they are degraded. Different modes of morphogen transport have been proposed (Wartlick et al., 2009; Müller et al., 2013; Kicheva and Briscoe, 2023), including diffusion (Stapornwongkul and Vincent, 2021), transcytosis (Kruse et al., 2004; Bollenbach et al., 2005; Romanova-Michaelides et al., 2022), and directed transport via cytonemes (Sanders et al., 2013) and by cell division following receptor binding (Farin et al., 2016).

The simplest and most common mode of morphogen transport is by diffusion through the embryonic extracellular space (ECS) (Crick, 1970; Lander et al., 2002; Yu et al., 2009; Harish et al., 2023). The ECS is the complex-shaped interstitial space between the cells, which resembles a porous medium (Rusakov and Kullmann, 1998; Nicholson and Syková, 1998). When circumventing obstacles in tortuous porous media, molecules have to move further, on average, to reach the same mean square displacement as in free space, leading to reduced effective diffusivity (van Brakel and Heertjes, 1974; Boudreau, 1996; Sbalzarini et al., 2005). Moreover, some parts of a porous medium may be inaccessible due to imperfect pore connectivity, i.e. islands and dead ends (Zhu et al., 2024), compartmentalizing the space and leading to morphogen accumulation in ECS cavities (Kuhn et al., 2022).

The diffusive tortuosity *τ*_d_ is the physical quantity commonly used to capture geometric hindrance to diffusion in a porous medium (Archie, 1942; Ghanbarian et al., 2013). It is defined as:
(1)

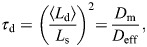
where *D*_m_ is the molecular diffusion coefficient of the morphogen in free solution, and *D*_eff_ is the effective diffusion coefficient in the porous medium. The ratio of the two diffusion coefficients is related to the average path length 〈*L*_d_〉 traveled by a diffusing molecule, compared to the straight-line distance *L*_s_ between the start and end points of that path.

In addition to hindering diffusion, anisotropic porous geometries also lead to nonlinear chemical reactions. Morphogen reactions include their secretion from localized source cells, interaction with extracellular matrix (ECM) components (i.e. non-receptor binding), such as heparan sulfate proteoglycans (HSPGs) (Forsten-Williams et al., 2008; Sarrazin et al., 2011; Stapornwongkul and Vincent, 2021), and receptor binding and endocytosis in the target tissue. These reactions depend on the cell surface area, ECS pore connectivity and ECS space volume.

As a diffusion-limited process, the effective morphogen-receptor binding rate can be significantly smaller than the intrinsic rate and can depend on the local ECS geometry (Weisz, 1973; Reeves et al., 2006). Diffusive hindrance (Müller et al., 2013), domain compartmentalization (Zhang et al., 2019), and (non-)receptor localization and concentration (Stapornwongkul et al., 2020) hence interact to influence morphogen gradient formation and maintenance in porous ECS geometries. While tortuous ECS geometries have been hypothesized for a long time to influence morphogen gradients (Müller et al., 2013), the pore-scale (i.e. cell-scale) 3D geometry has not been resolved by previous numerical models.

**Fig. 1.**
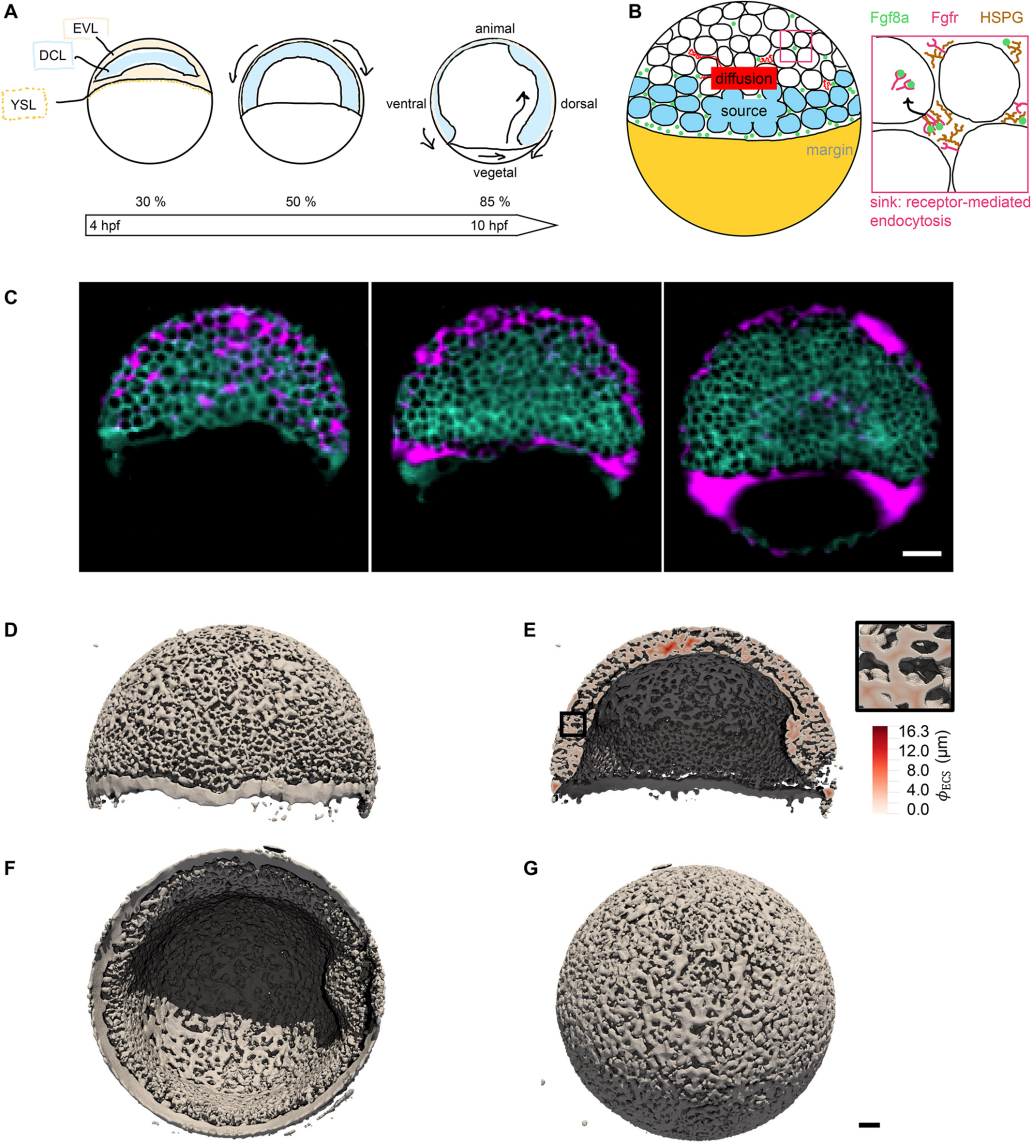
**The extracellular space resembles an anisotropic porous medium during zebrafish epiboly.** (A) Illustration of zebrafish epiboly. Representative stages at around 40%, 50% and 85% epiboly with deep-cell layer (DCL, blue), enveloping layer (EVL, yellow) and yolk syncytial layer (YSL, yellow dots). Starting at around 4 hpf (40% epiboly), the yolk moves toward the animal pole, and the blastoderm, which is composed of EVL, DCL and YSL, starts spreading over the yolk. Toward the end of epiboly, the blastoderm almost completely engulfs the yolk, and asymmetry along the dorsal-ventral axis emerges. (B) Scheme of Fgf8a gradient formation during zebrafish epiboly. Secreted by source cells at the blastoderm margin (blue), Fgf8a diffuses along tortuous paths through the ECS, further hindered by HSPG binding. The complex formation of Fgf8a, HSPG and Fgf receptors initiates internalization and endocytosis of Fgf8a into target cells acting as sinks (pink-framed box). (C) Light-sheet microscopy images of zebrafish epiboly. From left to right: exemplary optical sections from a Tg(bactin:hRas-EGFP) embryo at late blastula, early gastrula and mid-gastrula stages, respectively, following image acquisition using a light-sheet fluorescence microscope and multi-view reconstruction (see the section ‘Acquisition of light-sheet microscopy time-lapse video’). hRas-EGFP labels the cell membranes (green). ECS is marked by TMR-dextran injection (magenta). Unidirectional views and single optical sections are shown for simplicity. For all 25 timepoints, see Movie 1. (D-G) Visualization of the sparse-grid discretization of the ECS at ≈60% epiboly. (D) Side view oriented as in B and C. (E) Cross-sectional view with *φ*_ECS_ values in increasing distance to the ECS surface shown in shades of red (color bar). (F) Cross-sectional views from the vegetal pole. (G) View from the animal pole. Scale bars: 50 μm.

A morphogen that forms long-range concentration gradients in the ECS is fibroblast growth factor 8a (Fgf8a). As a member of the highly conserved Fgf family, Fgf8a is involved in maintaining the midbrain-hindbrain boundary, and in somitogenesis, limb induction and tissue patterning in zebrafish (Reifers et al., 1998; Moon and Capecchi, 2000; Draper et al., 2003; Scholpp et al., 2003). In particular, Fgf8a forms a gradient along the animal-vegetal (AV) axis during zebrafish epiboly (Yu et al., 2009; Harish et al., 2023). Epiboly is the first morphogenetic movement in zebrafish during embryogenesis. It starts ≈4 h post fertilization (hpf) when actomyosin contraction in the yolk syncytial layer (YSL) pulls the enveloping layer (EVL) towards the vegetal pole (Behrndt et al., 2012; Bruce, 2016; Morita et al., 2017), as depicted in Fig. 1A. This causes the blastoderm, consisting of the EVL and a deep-cell layer (DCL), to thin and spread over the yolk until completely engulfing the yolk by the end of epiboly (Behrndt et al., 2012; Bruce, 2016; Morita et al., 2017).

During this dynamic cell movement, Fgf8a is secreted by a band of cells at the blastoderm margin, from where it diffuses throughout the ECS (Scholpp and Brand, 2004; Yu et al., 2009; Harish et al., 2023), as illustrated in Fig. 1B. Interaction with HSPG at the cell surfaces and in the ECS is assumed to stabilize Fgf8a gradients by protecting Fgf8a from degradation and by slowing down its diffusion (Yu et al., 2009; Harish et al., 2023; Gupta et al., 2024). Cell-surface HSPGs also enhance Fgf8a binding to its receptors, Fgfr1 and Fgfr4, in the target tissue (Gupta et al., 2024). It is assumed that the internalized complex consists of Fgf8a, Fgfr and HS in a 2:2:2 stoichiometry (Schlessinger et al., 2000; Ornitz and Itoh, 2015). Complex internalization involves clathrin-mediated endocytosis. Together, these processes – secretion, diffusion, degradation and endocytosis – amount to a source-diffusion-degradation (SDD) (Gregor et al., 2007; Bollenbach et al., 2005; Kicheva et al., 2007) mechanism with additional HSPG binding (Yu et al., 2009; Harish et al., 2023).

Whether this SDD+HSPG mechanism is sufficient in realistic ECS geometries to explain the experimentally observed Fgf8a gradient, however, remains to be shown. It is difficult, if not impossible, to measure *in vivo* the individual roles of source and sink rates, HSPG binding, diffusion coefficient, and ECS geometry. To disentangle these different factors and quantify how they modulate morphogen gradient formation individually and collectively, mathematical and numerical models are required.

One-dimensional (1D) models have been successfully used to study morphogen gradient robustness to different source sizes (Dalessi et al., 2011) and to the absence of a sink (Coppey et al., 2007), and how robustness can be established by non-receptor binding (Lander et al., 2007; Lei and Song, 2010; Stapornwongkul et al., 2020) and transcytosis (Bollenbach et al., 2005). 1D models have been further used to study scaling across different embryo sizes (Fried and Iber, 2014; Werner et al., 2015; Aguilar-Hidalgo et al., 2018; Romanova-Michaelides et al., 2022), and local accumulation times (LATs) for different source (Berezhkovskii et al., 2011) and sink configurations (Bressloff, 2022), as well as tissue growth (Yang et al., 2019).

Extension of the LAT expression to radially symmetric 2D and 3D models has shown that relaxation kinetics differ depending on the model dimensionality (Gordon et al., 2013). 2D models have further been used to study Dpp gradient precision (Bollenbach et al., 2008), and the role of receptor trafficking (Kruse et al., 2004) and transcytosis (Bollenbach et al., 2005) in Dpp gradient formation, later extended to effective nonlinear transport equations with analytical expressions for concentration-dependent diffusion coefficient and degradation rate (Bollenbach et al., 2007).

Another way to simplify the system for deriving analytical solutions is through geometrical upscaling, i.e. collectively representing the tortuous diffusion paths by an effective average constant (Islam and Manzocchi, 2019). Numerical homogenization is a common upscaling technique that has been used to model morphogen gradient formation in the cortical region of the embryonic *Drosophila* syncytium (Sample and Shvartsman, 2010), enabling the derivation of an analytical mean-field expression for that system. Minimal models are often well suited to test general principles of gradient formation. By simplifying the geometry, challenges associated with geometric complexity and temporal deformation in realistic embryo geometries are avoided. However, such models do not capture the roles that ECS surfaces play in gradient formation.

Another purpose of models is to guide *in vivo* experiments. Experiments on embryos are expensive in terms of money, time and ethical considerations. A computational embryo, i.e. a numerical representation of some aspects of a real embryo, enables *in silico* experiments to inform and design *in vivo* experiments, often reducing their number. Computational experiments have the advantage of providing full parameter control. This enables disentangling intertwined factors, such as reaction rates and geometry. The more similar the computational and the real embryo, the lower the level of abstraction required to map between the experiments. In particular, nonlinearities, including anomalous diffusion (Hornung et al., 2005), and anisotropies of the embryo geometry are difficult to capture in simplified 1D or 2D models. 3D models based on experimental imaging data are required to test hypotheses about how morphogen gradients form in realistic embryo geometries.

Image-based models of embryonic development have been developed in 2D+time, e.g. to model limb growth and digit patterning during vertebrate development (Newman et al., 2008; Marcon et al., 2011; Lesnicar-Pucko et al., 2020 preprint; Millan Claro et al., 2022) and kidney branching morphogenesis (Menshykau et al., 2019), as well as in 3D+time to model mouse limb-bud growth (Boehm et al., 2010; Dalmasso et al., 2022). However, these image-based geometries consisted of a singly connected surface (or outline in the 2D models), differing considerably from the porous zebrafish ECS geometry with its many intricately shaped and disconnected surfaces. These ECS surfaces, mostly cell surfaces, play an important role in gradient formation by confining the diffusion space and localizing morphogen sources and sinks. Despite this significant role, the zebrafish ECS geometry has been simplified in models of morphogen gradient formation during epiboly to the arc of a circle (Yu et al., 2009; Müller et al., 2013) or to the surface of a sphere (Li et al., 2020a, 2022).

The challenge with modeling realistic ECS geometries is their computational cost, which can be prohibitive. As embryo geometries cannot be mathematically parameterized, they require algorithmic representations at high resolution to capture pore-scale dynamics from interstitial spaces (nanometers) to the embryo size (millimeters). Memory-efficient algorithms and acceleration of the multi-scale simulation on Graphics Processing Units (GPUs) are thus required to render simulations in realistic embryo geometries feasible.

Here, we derive realistic 3D ECS geometries of zebrafish epiboly from light-sheet microscopy volumes and represent them in a memory-efficient way using geometry-adaptive sparse grids (Incardona et al., 2021). In these image-based geometries, and using a GPU-accelerated algorithm (Stark and Sbalzarini, 2023), we simulate Fgf8a gradient formation by solving a system of coupled partial differential equations (PDEs), accounting for the spatial variations of sources and sinks, spatial heterogeneity of the geometry, and the interaction of Fgf8a with HSPG.

We use the resulting image-based computational embryo to test how different factors influence Fgf8a gradient shape, finding that the normalized gradient is robust against changes in the source and sink rates, whereas it is sensitive to changes that affect diffusive hindrance. Besides changes in the molecular diffusion constant of the morphogen, the latter includes changes in the extracellular HSPG concentration and changes to the ECS geometry. Concretely, peak concentrations near the source are more preserved, and the gradients are steeper and shorter for higher HSPG concentrations, lower diffusion coefficients and higher ECS tortuosity.

## RESULTS

We reconstruct realistic 3D ECS geometries based on light-sheet microscopy volumes of zebrafish epiboly, as described in the Materials and Methods. This includes image segmentation, numerical surface representation as a level-set signed-distance function, and geometry-adaptive discretization of the ECS using distributed sparse block grids. Exemplary optical sections of the light-sheet microscopy video are shown in Fig. 1C and Movie 1. A full 3D visualization of the image-based model is shown in Fig. 1D-G for the time point at ≈60% epiboly. We use this image-based model, simulation algorithm and the parameter values described in Materials and Methods to study the emergence, maintenance and robustness of the Fgf8a gradient in realistic ECS geometries of zebrafish embryos during epiboly. The computational model allows us to disentangle the influences of different molecular parameters and of the ECS geometry and shape. This enables testing for the sufficiency of an SDD mechanism of gradient formation and quantifying the sensitivity of the gradient to changes in the ECS geometry.

### A SDD+HSPG-binding mechanism is sufficient to generate *de novo* Fgf8a gradients

We simulate *de novo* Fgf8a gradient formation in reconstructed zebrafish ECS geometries at ≈40%, 60% and 75% epiboly. The simulations start from initial concentrations 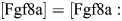 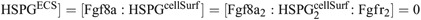 everywhere in the embryo to test whether a SDD+HSPG-binding mechanism is sufficient for the emergence of an AV gradient.

**Fig. 2.**
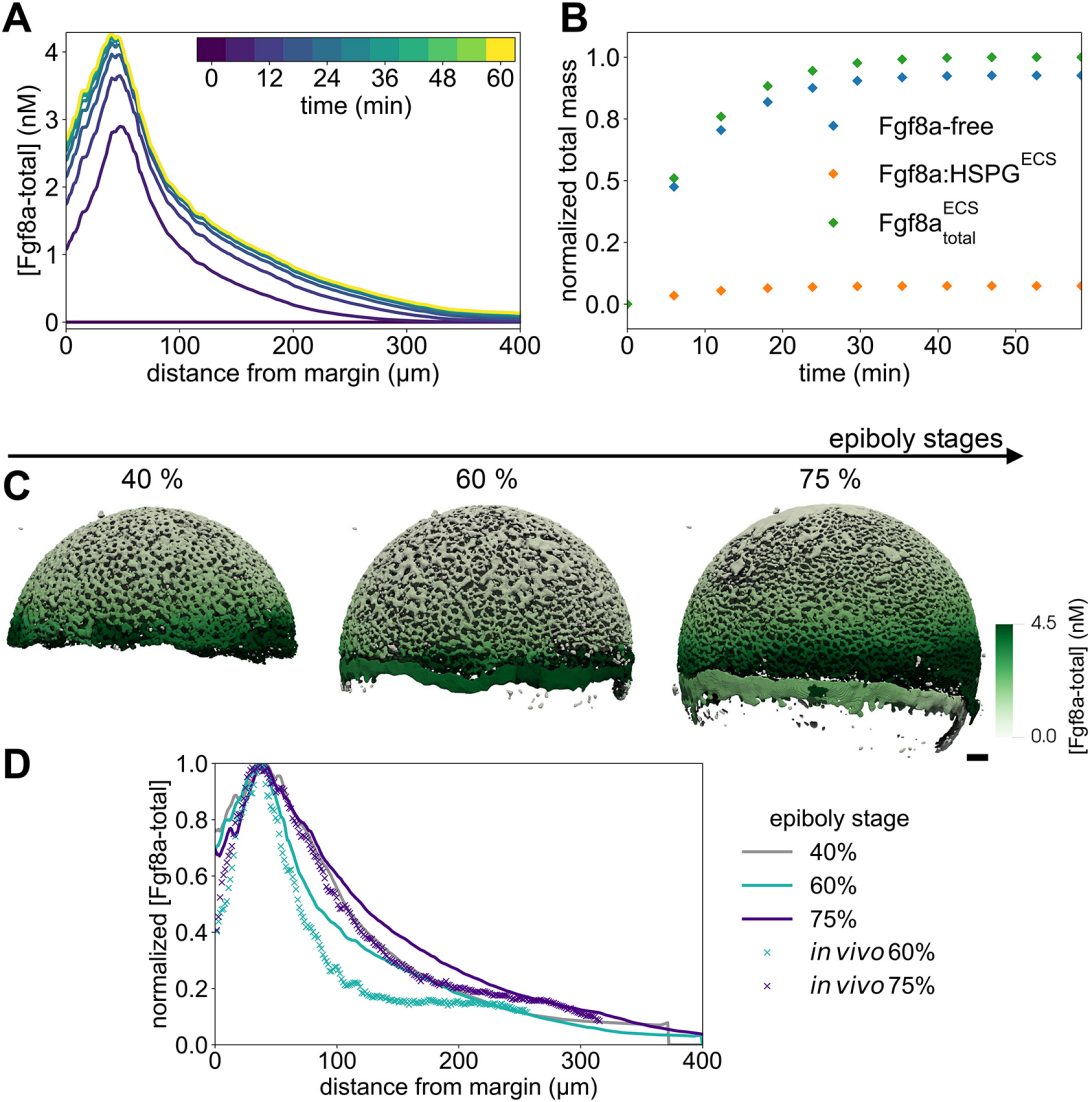
**A SDD+HSPG mechanism is sufficient to explain the formation of the Fgf8a gradient in realistic ECS geometries.** All Fgf8a concentrations in the simulation were initially set to zero throughout the embryo. (A) Evolution of the emerging AV gradient profile of (Fgf8a - total) over time (colored bar). The gradient reaches a steady state at ≈30 min. (B) The Fgf8a-free/Fgf8a:HSPG^ECS^ ratio remains almost constant (92.6%/7.4%) and close to the experimentally measured values (93%/7%) throughout the 60 min of simulated time. (C) Visualization of the simulated Fgf8a-total concentrations in the ECS geometries at 40%, 60% and 75% epiboly. (D) Simulated (lines) and experimentally measured (symbols) AV profiles at different stages (see key). Experimental profiles were only available at 60% and 75% epiboly, and show the mean over *N* = 15 embryos. Simulated profiles are computed at 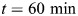. Scale bar: 50 μm.

The resulting concentration profiles and total mass of extracellular Fgf8a over time at 60% epiboly are shown in Fig. 2A and B, respectively. A steady state is reached at ≈30 min. The Fgf8a/Fgf8a:HSPG^ECS^ ratio remains almost constant (92.6%/7.4%) throughout the simulated 60 min and very close to the *in vivo* measured ratio (93%/7%). Overall, the simulated steady-state profile is similar to the experimental profile (see Fig. 7A) but shows a flatter gradient.

The simulated gradient is best described by two exponential functions, one for the first part between *d*=35 μm and *d*=125 μm with decay length 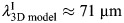, and one for the second part between *d*=125 μm and *d*=400 μm with 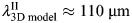. The decay length in the first part is closer to the *in vivo* decay length of *λ*_in vivo_≈50 μm, whereas the second part is more similar to the gradient of a 1D model (see Fig. 14A). A possible reason for this difference is that the *in vivo* profile has been averaged over a sub-volume of the embryo (yellow dotted lines in Fig. 7A), whereas the simulated profile is computed across the entire ECS. Another reason could be modeling errors in the ECS geometry reconstruction or the simulation parameters. Indeed, the image segmentation tends to overestimate ECS thickness, reducing geometrical hindrance in the model compared to the real embryo and enabling Fgf8a to diffuse faster and further, which would explain the gradient flattening and increased decay length.

A particular characteristic of the *in vivo* Fgf8a gradient that could not be clarified experimentally so far is the kink near the source region. The image-based model reproduces this kink. As the source rate in the model is constant in space and time, this zonation effect is likely caused by ECS geometry heterogeneities leading to effective compartmentalization of the source region. This is confirmed by computing the ECS porosity profile along the AV axis (see Fig. 12A), revealing that the porosity is lowest at the margin. This leads to a higher diffusive hindrance at the margin, which, adding to the smoothing effect of an extended (i.e. not point) source (Dalessi et al., 2011), could explain the kink in the source region. This hypothesis is further supported by the fact that the kink could be partly recovered in a 1D model by upscaling the porous source, i.e. representing it by an effective average constant along the AV axis (see Materials and Methods; Fig. 12C).

We repeated the *de novo* gradient formation simulation in ECS geometries at ≈40 and ≈75% epiboly. The results are shown in Fig. 2C and D, respectively. Again, the simulated profiles are slightly flatter than the experimentally measured ones but are overall similar and show the same trend over developmental stages. In addition, in the model, propagation lengths increase as epiboly progresses, probably due to a widening of the source band.

Interestingly, in addition to the AV gradient, the simulation also reproduces the DV gradient observed *in vivo* at 75% epiboly (Fig. 11B,C). Since, in the model, the rates of all sources, sinks and HSPG binding are invariant along the DV axis, this gradient can be interpreted as a consequence of ECS geometry asymmetry. Indeed, we find that the reconstructed ECS geometry has a porosity gradient along the DV axis with lower porosities (and therefore potentially higher morphogen retention) towards the ventral side (see Fig. 11D). In addition to this sufficient geometric asymmetry, earlier patterning events have been proposed to contribute to DV gradient formation (Fürthauer et al., 1997; Reifers et al., 1998).

Taken together, these results show that the SDD+HSPG-binding mechanism modeled here is sufficient to cause the emergence of AV and DV gradients in zebrafish embryo ECS geometries during epiboly. The mechanism also maintains the experimentally measured Fgf8a population fractions and explains the gradient evolution across epiboly stages.

### Fgf8a normalized gradients are robust to changes in rate constants but sensitive to changes in ECS geometry

Factors such as temperature and DNA distribution can cause fluctuations in gene expression levels and therefore reaction rates. Gradient robustness is the capacity to buffer the effect of such fluctuations and is important to ensure that tissues develop correctly over a range of environmental conditions. Using our model in ECS geometries at 60% epiboly, we quantify how the rates of sources, sinks, HSPG binding and diffusion influence the gradient profile. In addition, we study the impact of ECS geometry on the gradient.

#### The normalized gradient profile is robust against changes in source and sink rates

Morphogen gradients are surprisingly robust against changes in the source rate (Eldar et al., 2002, 2004; Zhang et al., 2019; Madamanchi et al., 2021). In previous models, this robustness has been explained by nonlinearities of the ligand flux when the morphogen is transported by transcytosis (Bollenbach et al., 2005) or by nonlinear, self-enhanced degradation in a free-diffusion model (Eldar et al., 2003). This is because in nonlinear degradation, the sink rate changes with morphogen concentration, buffering the effect of varying morphogen production on the gradient amplitude (Eldar et al., 2003; Bollenbach et al., 2005). For linear degradation rates, however, the gradient amplitude is expected to be proportional to the source rate (Wartlick et al., 2009; Gonzalez-Gaitan and Jülicher, 2014; Aguilar-Hidalgo et al., 2018).

To test how the source rate influences gradient formation in realistic ECS geometries, we simulate *de novo* Fgf8a gradient formation for different *k*_source_, keeping all other parameters fixed, until a steady state is reached at *t*_max_=60 min. The resulting AV gradient profiles in Fig. S4A show that higher *k*_source_ lead to higher overall concentration levels. The gradient amplitude is proportional to *k*_source_ (see Fig. S4E), as expected for linear degradation (Wartlick et al., 2009; Gonzalez-Gaitan and Jülicher, 2014; Aguilar-Hidalgo et al., 2018).

**Fig. 3.**
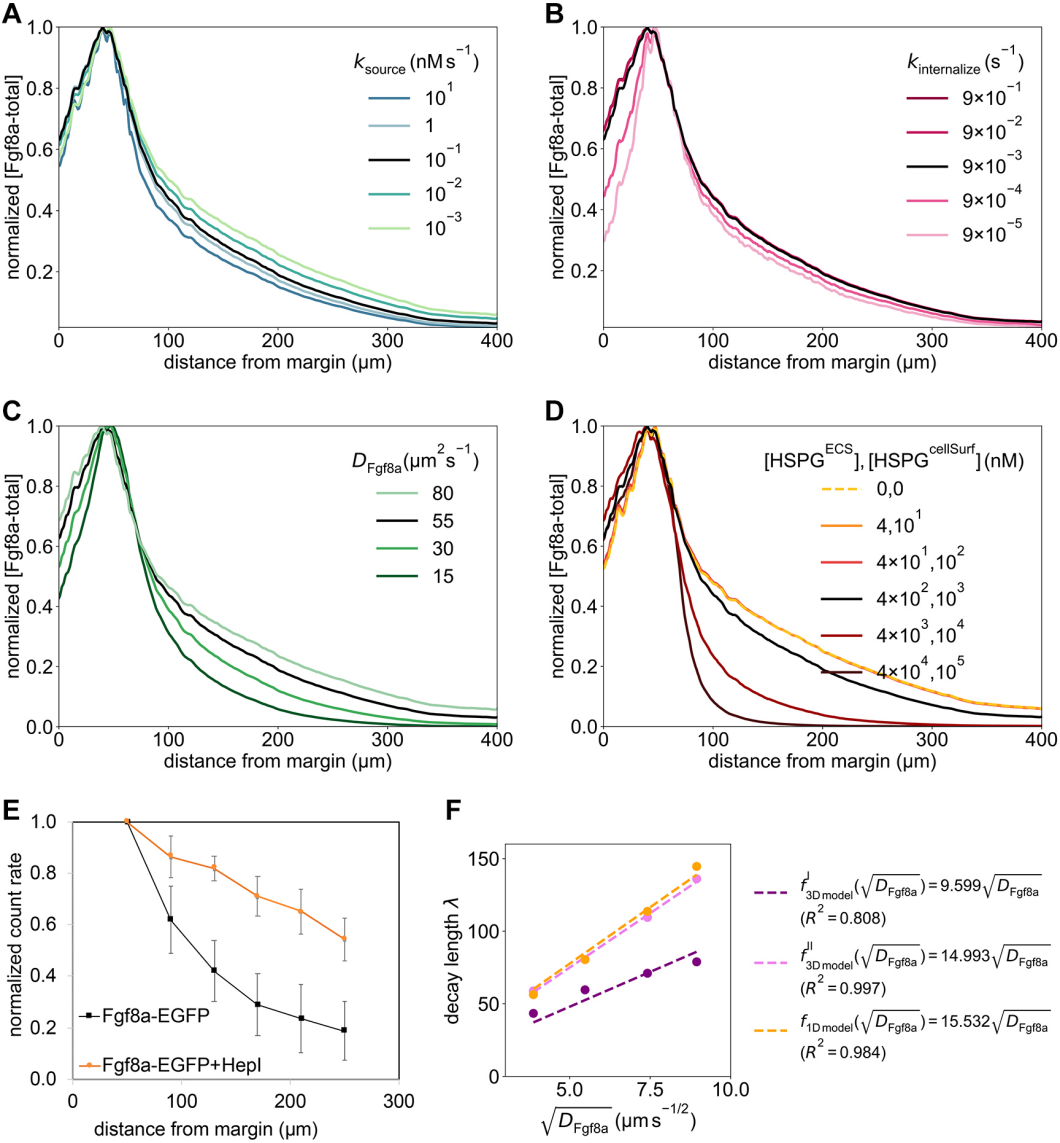
**Sensitivity of the simulated steady-state Fgf8a gradients to variations in the model parameters and *in vivo* gradient sensitivity to HepI injection.** (A,B) The normalized simulated gradient shows remarkable robustness against changes in source (A) and sink rates (B) over four orders of magnitude (see key). (C,D) Changes in the effective diffusivity, by changing either the molecular diffusion coefficient of Fgf8a (C) or HSPG concentrations (D), strongly affect the gradient. The higher the HSPG binding and the smaller the diffusion coefficient, the steeper and shorter the gradient. The baseline gradient (see the section ‘A SDD+HSPG-binding mechanism is sufficient to generate *de novo* Fgf8a gradients’) is shown as a solid black line in all panels. All profiles are normalized by their respective maximum *xz*-plane-averaged concentration. Profiles are shown at 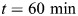. (E) FCS count rates of Fgf8a without (black squares) and with (orange circles) HepI injection. HepI cleaves off the HS side-chain of HSPGs. The Fgf8a gradient of HepI-injected embryos is shallower. Reproduced, with permission, from Harish et al. (2023). *N*=20 embryos for control, *N*=13 for HepI; data are mean±s.d. (F) The gradient decay lengths *λ* scale linearly with the square root of the molecular diffusion coefficient of the morphogen, as expected for gradients forming by an SDD mechanism (Kicheva et al., 2012; Hidalgo et al., 2019 preprint). The linear fits show the least-squares solutions obtained using numpy.linalg.lstsq (Harris et al., 2020). The key provides the fitted proportionality coefficients and the respective goodness of fit *R*^2^.

Normalizing each profile by its respective maximum concentration reveals that the gradient shape is almost invariant to changes in *k*_source_ (see Fig. 3A). This is in agreement with theoretical results, in which 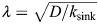 is independent of *k*_source_ (Wartlick et al., 2009). Compared to the baseline *k*_source_=0.1 nM s^−1^, the gradient becomes only slightly flatter when decreasing *k*_source_ and slightly steeper when increasing *k*_source_. These deviations are smaller than when solving the same equations in a 1D domain (see Materials and Methods and Fig. 13A). Another difference between the 3D and the 1D model is that the proportionality coefficient of the gradient amplitude with respect to *k*_source_ is almost four times lower in 3D than in 1D (Fig. S4E). This means that, in the porous 3D geometry, absolute gradient amplitudes are almost four times more robust against varying *k*_source_ than in 1D. This can be explained by the fact that in the 3D model, the sources are restricted to the cell surfaces and fragmented by the porous geometry, leading to a lower effective *k*_source_, as in the 1D model. Another explanation could be that the effective degradation in the 3D model is higher, as the gradient amplitude is inversely proportional to the effective sink rate (Wartlick et al., 2009).

For the sink rate, *in vivo* observations suggest that the gradient is not robust. It has been shown that modulating Fgf8a endocytosis in live zebrafish embryos makes normalized gradients shallower when inhibiting endocytosis and steeper when upregulating endocytosis (Yu et al., 2009). We test the behavior of our model by changing *k*_internalize_ while keeping all other parameters unchanged. The resulting gradient profiles in Fig. S4B suggest that decreasing *k*_internalize_ leads to higher concentration levels, which is an expected consequence of reduced morphogen degradation (Gonzalez-Gaitan and Jülicher, 2014; Aguilar-Hidalgo et al., 2018). Normalizing the profiles, however, it can again be seen in Fig. 3B that the gradient shape is almost unaffected by changing *k*_internalize_ across four orders of magnitude. A significant difference can only be seen within 50 μm of the margin, where the gradient becomes steeper for smaller *k*_internalize_ than the baseline *k*_internalize_=9×10^−3^ s^−1^, but only slightly flatter for *k*_internalize_ larger than baseline. The robustness of the normalized gradient against variations in *k*_internalize_ still holds for 10^3^ times larger *k*_complex_, 10^3^ times larger *k*_on_ and 10^3^ times smaller *k*_off_, as shown in Fig. S6A,C,E. The gradient amplitude, however, is more sensitive to changes in *k*_internalize_ for larger *k*_on_ and *k*_complex_, and smaller *k*_off_ (Fig. S6B,D,F). A possible explanation for this is that, in our model, HSPG binding precedes complex formation and internalization so that higher HSPG affinities can increase the sensitivity to internalization rates.

The robustness of the gradient shape against changes in *k*_internalize_ seems to be independent of ECS geometry, as the same results are obtained in the 1D model (Fig. 13B). In addition, the gradient there is still robust for 10^3^ times larger *k*_on_ and *k*_complex_, as well as for 10^3^ times smaller *k*_off_ (see Fig. S8A,C,E). The same robustness is obtained when varying *k*_complex_, as shown in Fig. S5A for the 3D and Fig. 13G for the 1D model. For increasing *k*_complex_, the absolute concentrations are maintained at similar levels (see Fig. S5B and Fig. S7G), which could be explained by the fact that complex formation protects Fgf8a from molecular degradation, counterbalancing its decay in the ECS.

The simulation does therefore not explain the experimentally observed sensitivity of the gradient to endocytosis rates. The gradient flattening observed *in vivo* is therefore not caused by the molecular mechanisms modeled here. Instead, we speculate that it could be caused by side effects of a dominant-negative mutation of dynamin, a GTPase that pinches off endocytic vesicles from the plasma membrane. Changes in dynamin function are therefore not specific to Fgf8a endocytosis, and may also affect other feedback loops and downstream signaling cascades.

In conclusion, the simulation results show that a SDD+HSPG mechanism is sufficient for generating gradients whose normalized profile is robust to changes in *k*_source_ and *k*_internalize_ across four orders of magnitude. However, this robustness does not transfer to the absolute concentration gradient, the overall levels of which increase for increasing *k*_source_ and decreasing *k*_internalize_.

#### Lower diffusion coefficients and higher HSPG binding rates lead to steeper and shorter gradients

The intrinsic molecular diffusion constant of a morphogen was measured by fluorescence correlation spectroscopy (FCS) (Harish et al., 2023). The effective diffusion coefficient in the porous ECS, however, depends on the spatially heterogeneous tortuosity and on interactions with ECM molecules. The present image-based model enables the determination of the effective diffusion coefficient (Sbalzarini et al., 2005) and the testing of how different molecular diffusion coefficients affect the resulting morphogen gradient.

To achieve this, we simulate *de novo* gradient formation using different molecular diffusion coefficients of Fgf8a-free. The results are shown in Fig. S4C (absolute values) and Fig. S3C (normalized). Compared to the baseline diffusivity of *D*_Fgf8a_=55 μm^2^ s^−1^, lower diffusion coefficients result in steeper gradients and shorter propagation lengths, with narrower higher peaks at the source. Increasing the diffusion coefficient leads to gradient flattening, longer propagation and lower peak concentration.

As shown in Fig. 3F, and detailed in the Materials and Methods and in Fig. 14B,C, the decay length in the second half of the simulated gradient of the image-based model, 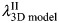, and the decay length of the 1D model, *λ*_1D model_, are both proportional to 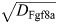, as expected for morphogen gradients forming by a SDD mechanism (Kicheva et al., 2012; Hidalgo et al., 2019 preprint). The proportionality factor is 15 for the 3D model (*R*^2^=0.997) and 16 for the 1D model (*R*^2^=0.984). The decay length in the first half of the gradient of the 3D model, 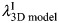, with respect to 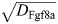, however, has a lower coefficient of determination (*R*^2^=0.808, proportionality factor of 10). Since this region includes the source, this nonlinearity could be explained by the shape of the source (Dalessi et al., 2011).

The dependence of the gradient profile on the molecular diffusion coefficient implies that embryos can generate distinct but overlapping gene expression patterns by using morphogens with different diffusion coefficients. Faster diffusing morphogens can propagate further and have longer signaling ranges in the embryo, whereas slower diffusing morphogens maintain higher concentrations near the source and act more locally.

In addition to different intrinsic diffusion coefficients, morphogens of similar free diffusivities can have different effective diffusivities when differently hindered in their diffusion by (transient) ECM and receptor binding, as it has been shown, e.g. by single-molecule tracking of Nodal and Lefty in the zebrafish ECS (Kuhn et al., 2022). To test the influence of morphogen binding by HSPG on gradient formation, we simulate *de novo* gradient formation for different [HSPG^ECS^] and [HSPG^cellSurf^]. As shown in Fig. 3D, higher HSPG concentrations lead to gradients that are considerably steeper with shorter propagation lengths. This is in line with previous works in which binding to immobile or slowly diffusing non-receptors shortened the gradient (Stapornwongkul et al., 2020). Similarly, increasing *k*_on_ leads to shorter and steeper gradients (see Fig. S5C), whose absolute concentration levels are highest for the baseline value, lowest for 10 times larger *k*_on_, and in between these values for 10^2^−10^4^ times larger *k*_on_ (see Fig. S5D). Increasing HSPG binding by reducing *k*_off_ does not affect the normalized profile but leads to higher gradient amplitudes (Fig. S5F). Similar to *k*_complex_, this could be explained by HSPG binding protecting Fgf8a from proteolysis. We find the same sensitivity to changes in HSPG binding in the 1D model (see Fig. 13D-F and Fig. S7D-F). HSPG therefore acts on the gradient by modulating the effective morphogen diffusivity through transient matrix binding.

Flatter Fgf8a gradients for lower HSPG concentrations are in agreement with *in vivo* HepI-injection experiments. HepI is an enzyme that cleaves the HS side chains of HSPG, reducing the slow-diffusing Fgf8a fraction, thereby causing a shallower gradient (Yu et al., 2009; Harish et al., 2023) as measured by FCS and shown in Fig. 3E. HepI injection also increased the overall Fgf8a concentration in the ECS, which has been explained by Fgf8a dissociating from cell-surface HSPGs (Harish et al., 2023). Our simulations reproduce this observation, showing higher Fgf8a peak concentrations at lower HSPG concentrations (see Fig. S4D).

Interestingly, decreasing HSPG concentrations below [HSPG^ECS^]=40 nM and [HSPG^cellSurf^]=100 nM does not further flatten the gradient. Even when setting all HSPG concentrations to zero and modeling gradient formation by an SDD mechanism, the same profile emerges as for [HSPG^ECS^]=40 nM and [HSPG^cellSurf^]=100 nM (see coinciding lines in Fig. 3D). The same is observed when increasing *k*_off_ (Fig. S5E). This suggests that a minimal level of HSPG binding is required for any relevant effect on the gradient. This makes sense because, below the baseline, the freely diffusing Fgf8a fraction constitutes >92% of the total Fgf8a, dominating the gradient profile. This also shows that, under the current modeling assumptions, a SDD mechanism is sufficient for the formation and maintenance of some gradient, which is, however, flatter than the baseline and the *in vivo* gradient.

Both *in vivo* and *in silico*, HSPG^cellSurf^ is involved in receptor binding and endocytosis of Fgf8a. Decreasing [HSPG^cellSurf^] thus also decreases sink fluxes. Whereas changing the sink rate *k*_internalize_ did not affect the normalized simulated gradient (Fig. 3), reducing the effective sink flux does.

In conclusion, the present results show that morphogen gradients are sensitive to changes in morphogen diffusivity. Both reducing the molecular diffusion coefficient and the diffusive hindrance by HSPG binding have a similar effect, leading to steeper gradients with shorter range and higher peak near the source. This is in agreement with previous 1D models showing that lower diffusion coefficients lead to steeper and shorter gradients (Wartlick et al., 2009) and with *in vivo* experiments showing that HSPG binding is crucial for maintaining high concentrations near the source and for restricting gradient propagation lengths (Harish et al., 2023).

#### Tortuous ECS geometries stabilize Fgf8a gradients

The effect of decreasing HSPG concentrations is much smaller than when increasing them (Fig. 3D). Indeed, the gradient remains almost unchanged when reducing [HSPG^ECS^] or [HSPG^cellSurf^] by more than 10-fold. This suggests that the diffusive hindrance from HSPG binding is dominated by the tortuosity of the ECS geometry. This is consistent with HSPG being mostly localized near cell surfaces, mirroring the ECS geometry.

We use our realistic 3D ECS geometry model to quantify the effect of diffusive tortuosity on Fgf8a gradients. For this, we perturb the modeled ECS geometry and quantify the influence of these perturbations on simulated Fgf8a gradients. Such perturbations are straightforward in the numerical description of the ECS as a signed-distance function. Changing the surface level-set from the standard *φ*_ECS_=0 to other values uniformly changes the ECS tube diameters and thus the ECS density, connectivity and volume.

**Fig. 4.**
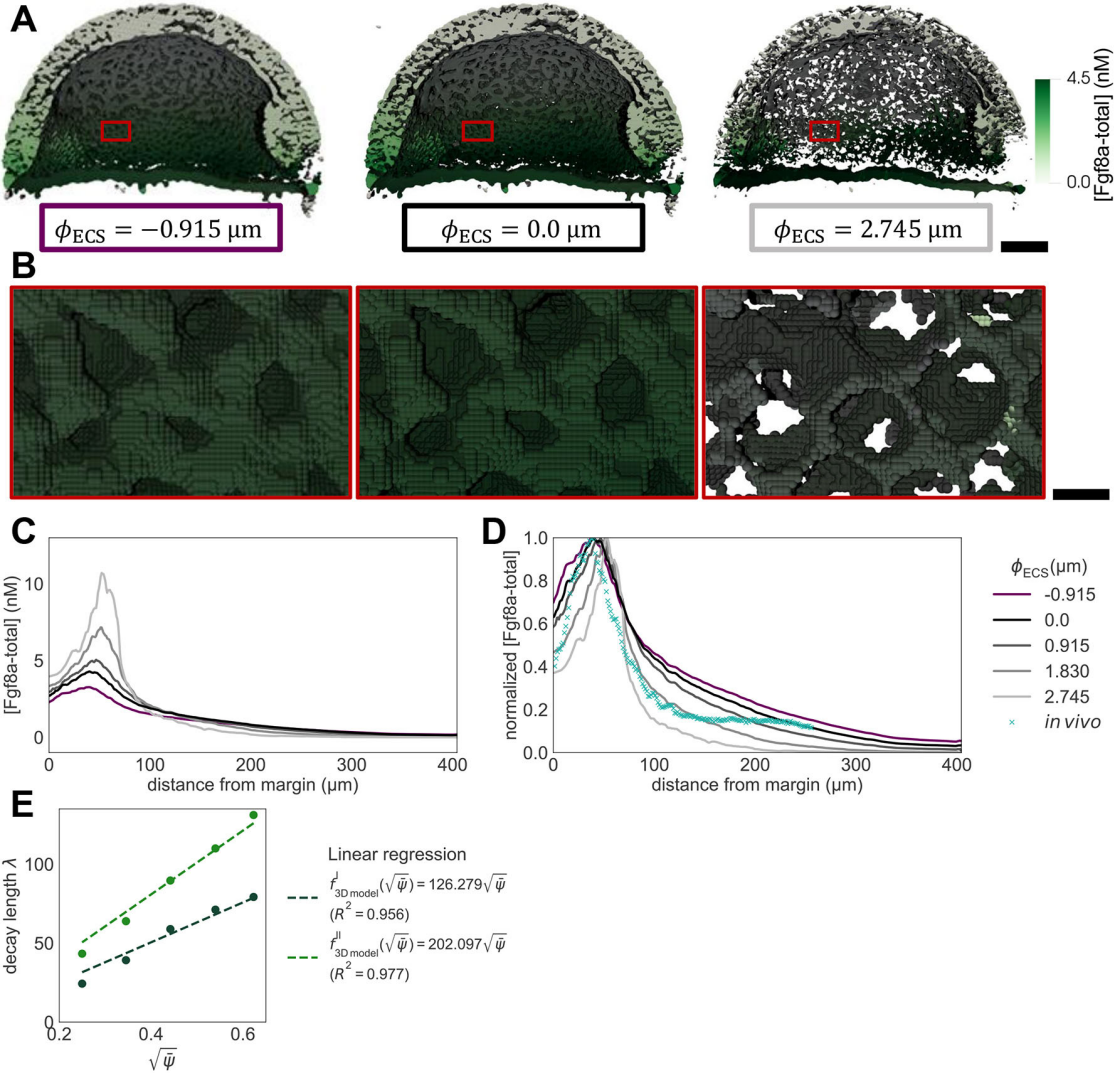
**ECS geometry controls Fgf8a gradient steepness and range.** To test how ECS tortuosity influences Fgf8a gradients, we simulated *de novo* gradient formation for different ECS geometries. (A) Clipped visualizations of the baseline ECS geometry (center) and two perturbed versions (left and right) with simulated Fgf8a concentrations overlaid in green, scaled to 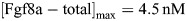 (see color intensity bar). The ECS tube thickness is perturbed by changing the boundary location along *φ*_ECS_: shifting down by one grid spacing *h*=0.915 μm (2 pixels), i.e. *φ*_ECS_=− 0.915 μm, to obtain thicker tubes; and increasing by 3*h*, i.e., *φ*_ECS_=2.745 μm to obtain thinner tubes. (B) Higher magnification images of the areas outlined in A. (C) Absolute AV concentration profiles at 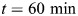. Colors correspond to different geometries (see key). Compared to the baseline geometry (solid black line), decreasing ECS tube sizes limits Fgf8a propagation lengths and leads to a steeper gradient with a higher peak (gray lines), whereas increasing the ECS tube size leads to a flatter gradient (violet line). (D) The same profiles normalized to their respective maximum concentration values. The *in vivo* profile at 60% epiboly (symbol) is more similar to the simulated profile for thinner tubes of *φ*_ECS_=1.830 μm, the average decay length of which, 
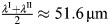
, is close to the *in vivo* decay length λ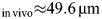. (E) Linear scaling of *λ*^I^ and *λ*^II^ with respect to 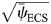. The linear fit is the least-squares solution obtained using numpy.linalg.lstsq (Harris et al., 2020). The key provides the fitted proportionality coefficients and their goodness of fit *R*^2^. Scale bars: 100 μm (A); 10 μm (B).

We vary the geometry in steps of *h* from *φ*_ECS_=3*h*=2.745 μm (thinner ECS, higher tortuosity) down to *φ*_ECS_=− 1*h*=− 0.915 μm (thicker ECS, lower tortuosity) in comparison with the baseline *φ*_ECS_=0. The unit of length *h*=0.915 μm is the spacing of the numerical grid, i.e. twice the pixel size of the light-sheet microscopy images. Examples of resulting perturbed geometries are shown in Fig. 4A,B for *φ*_ECS_=− 1*h* and *φ*_ECS_=3*h*. For these perturbed geometries, we compute steady-state Fgf8a gradient profiles, keeping all model parameters at their default values, as given in Table 2.

The resulting absolute concentration profiles in Fig. 4C show that the thinner the ECS tubes, the more elevated the peak near the source, the steeper the gradients and the shorter the range. The steeper shorter gradients for higher ECS tortuosities are also confirmed in the normalized profiles (Fig. 4D). Thickening the ECS tubes flattens the gradient. This is because the thicker the tubes, the lower the hindrance of morphogen diffusion. The decay lengths *λ*^I^ and *λ*^II^ of the normalized concentration profiles are proportional to the square root of the average ECS porosity 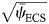 (Table 1, Fig. 4E and Fig. 14D), where 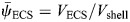. This means that *λ* is related similarly to 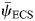 as to *D*_Fgf8a_, suggesting that 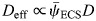, in agreement with previous works on inanimate porous media (Archie, 1942; Wakao and Smith, 1962; Etancelin et al., 2020).

**Table 1.** Relationship between decay length and ECS porosity

*φ*_ECS_ (μm)	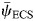	*λ*^I^ (μm)	*λ*^II^ (μm)
−0.915	0.39	79.24	131.14
0.0	0.29	71.19	109.98
0.915	0.20	58.90	89.65
1.830	0.12	39.21	63.90
2.745	0.06	24.34	43.33

Varying the ECS boundary location along *φ*_ECS_, and thereby the resulting average ECS porosity 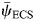, leads to changes in the decay length of the first half of the gradient, *λ*^I^, and to changes in decay length of the second half of the gradient, *λ*^II^.

The decay length of the *in vivo* gradient at 60% epiboly 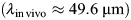 is matched best by the average decay length 
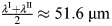
 of the ECS geometry whose tubes have been thinned by 1.830 μm compared to the baseline. These results show that morphogen gradients are sensitive to ECS porosity and that geometric hindrance is sufficient to tune morphogen gradient decay lengths.

The image-based model accounts for the diffusive tortuosity *τ*_d_ by explicitly resolving pore-scale ECS geometries. We next asked whether the effect of diffusive hindrance on gradient formation could be represented by a global *τ*_d_ in an upscaled (i.e. coarse-grained) model. To test this, we determined *τ*_d_ of the ECS at 60% epiboly by simulating a fluorescence recovery after a photobleaching (FRAP) experiment, finding *τ*_d_=2.5 (see Materials and Methods; Fig. 9). Using this *τ*_d_ to upscale the diffusion coefficient in a model of a spherical shell, recovered gradient steepness and range (see Fig. 10). However, this does not apply to the gradient kink at the source observed *in vivo*, which is reproduced by the pore-scale model but lost in the average upscaling. This shows that effective coefficients can only partly account for the geometric effects of porous ECS geometries as they do not consider asymmetries of the embryo geometry.

Taken together, these results show that the tortuous ECS geometry plays an important role in establishing and maintaining robust morphogen gradients. The geometric tortuosity regulates source peak height, gradient steepness and propagation length. In geometries of insufficient tortuosity, gradients are not maintained. Regulating gradient formation and maintenance therefore involves adjusting extracellular HSPG concentrations and ECS tortuosity to the morphogen diffusion coefficient. The embryo can thus control the morphogen gradient by tuning HSPG secretion and the shape of the interstitial space, e.g. by regulating cell adhesion and tissue rigidity (Mongera et al., 2018; Petridou et al., 2019).

## DISCUSSION

Combining image-based 3D geometry modeling with geometry-adaptive numerical simulation methods enabled the reconstitution of morphogen gradient emergence and maintenance *in silico* in realistic embryo geometries with fully resolved interstitial space, surface and bulk biochemistry. We reconstructed pore-scale extracellular space (ECS) geometries of zebrafish embryos during epiboly from light-sheet microscopy videos and numerically solved the governing equations of Fgf8a gradient formation in those geometries. The simulations were validated against *in vivo* fluorescence intensity profiles and single-molecule fluorescence correlation spectroscopy (FCS) measurements, showing that the *in vivo* gradient can be explained by a source-diffusion-degradation (SDD) mechanism with additional heparan-sulfate proteoglycan (HSPG) binding in the extracellular matrix.

We found that the decay length of the simulated Fgf8a gradient is robust against changes in the Fgf8a source and sink rates but sensitive to perturbations of the ECS geometry, suggesting that the geometry plays an important role in gradient shaping and maintenance. This indicates that the ‘SDD+HSPG+geometry’ mechanism proposed here is sufficient to explain the emergence and robustness of morphogen gradients. Comparing gradient formation in the porous model with a 1D model resulted in a similar sensitivity to most parameter changes except the source rate, for which the porous model was more robust. Source robustness has been previously explained by nonlinearities in morphogen degradation (Eldar et al., 2003; Bollenbach et al., 2005). Our results suggest that the porous ECS geometry contributes to this robustness by reducing effective source rates, as well as heterogeneous (non-)receptor distributions and morphogen retention through geometrical hindrance, buffering the source rate effects on the gradient.

We show that gradient steepness and range are regulated by diffusive hindrance through HSPG-binding and tortuous ECS geometries. The embryo can thus control gradient shape by tuning heparan-sulfate affinity and by modulating the HSPG secretion rate. In addition, ECS tortuosity can be controlled by modulating cell density and adhesion, varying the interstitial space. Modulation of cell adhesion can be observed, for example, during tissue fluidization of the central deep cells at the animal pole during the onset of doming (Mongera et al., 2018; Petridou et al., 2019). The aggregate diffusive hindrance is key to preserving high morphogen concentrations near the source and regulating gradient steepness and range.

Whereas it is well known that slower diffusion leads to steeper and shorter gradients (Kicheva et al., 2007; Wartlick et al., 2009), the question of how much those gradients differ for morphogens of different diffusion rates has not been shown in realistic tissue geometries before. In a 1D model, we can only compare effective average diffusion coefficients, which are hard to measure and might change over the course of embryogenesis as the tissue geometry or the receptor binding change (Stapornwongkul and Vincent, 2021). By explicitly resolving ECS geometries, the present approach does not require knowledge of effective diffusion coefficients but can be used to directly compare intrinsic diffusion coefficients, as measured by FC(C)S (Yu et al., 2009; Harish et al., 2023). The image-based model therefore provides a way of testing whether differences in diffusion coefficients alone are sufficient to explain the different gradient shapes observed for different morphogens.

Our results suggest that the ECS in the developing embryo effectively acts as a porous medium toward the morphogen gradient. When coarse-graining (i.e. computationally upscaling) the average porous-media characteristics of the ECS geometry into a spherical shell, the offset of the concentration peak near the blastoderm margin, reproduced in the fully resolved realistic ECS geometry, was naturally lost in an average upscaling.

Homogenization techniques could improve the upscaled model in the future. Homogenization by the multiscale expansion method has been used to derive analytical equations for modeling morphogen gradients in *Drosophila* syncytium (Sample and Shvartsman, 2010) and nutrient diffusion and uptake in biofilms (Dalwadi et al., 2018; Dalwadi and King, 2020). Instead of an analytical solution, a numerical upscaled 3D model could be used to consider spatial heterogeneities. The present fully resolved model could be used to validate and calibrate such an upscaled model.

The dependence on local geometric detail implies that our results are sensitive to imaging, image-segmentation and geometry-modeling errors. Although the image-based geometry used here was sufficiently realistic to demonstrate the pore-scale effects of geometry and explain the experimental observation, it likely underestimated real ECS tortuosity. The model could be refined in the future by enhancing image acquisition and segmentation.

The present model also neglects flows in the ECS due to tissue growth and cell motion, assuming that diffusive transport in the ECS is significantly faster than the time scale of tissue deformation. This is an approximation, as the real embryo geometry varies continuously over time. Although dilution effects are likely negligible during epiboly, as the ECS volume does not change (see supplementary Materials and Methods; Fig. S1), cell movement has been proposed to contribute to gradient formation by additional advective transport (Li et al., 2022), and coupling between morphogen reactive-diffusive transport and poroelastic tissue deformation has been proposed as an alternative mechanism for Turing instability to explain pattern formation (Recho et al., 2019). Quantifying the contributions from advection and deformation would require explicitly modeling tissue dynamics (Popović et al., 2017; Jülicher et al., 2018). Deriving continuous deformation maps requires image data with a higher time resolution than the light-sheet microscopy video used here. Once available, such deformation maps could be included in our model by correspondingly evolving the level-set function (Bergdorf and Koumoutsakos, 2006) and simulating the induced flows in the ECS (Schulze et al., 2024). Such simulations would also enable studying how morphogen gradients scale across different tissue sizes during growth (Fried and Iber, 2014; Werner et al., 2015; Aguilar-Hidalgo et al., 2018; Čapek and Müller, 2019).

The presented open-source modeling workflow and computer simulation algorithm are general and transferable, providing a more general method for building image-based computational embryos also for other model systems. Apart from morphogen gradients, regulation by diffusive hindrance from binding reactions and geometric tortuosity applies to other biological reaction-diffusion processes in complex tissue geometries, including bone repair (Li et al., 2020b), wound healing (Ben Amar and Wu, 2014), tumor cell proliferation (Ferreira et al., 2002) and therapeutics diffusion through the brain ECS (Wolak and Thorne, 2013). On the other hand, pathological conditions that cause cell swelling, e.g. during ischemia, increase the diffusive tortuosity, hindering the diffusion-mediated supply of those tissues (Hrabetová et al., 2003). These processes are – like morphogen gradient formation – modulated by the porous media characteristics specific to each tissue type, particularly the pore-scale tissue geometry.

## MATERIALS AND METHODS

We state the equations modeling Fgf8a gradient formation and discuss the numerical method and parallel computing software used to numerically solve these equations. We describe image acquisition, image processing and image-based reconstruction of the zebrafish ECS. We then provide details about the parameter search and the determination of porosity profiles and gradient decay lengths. Finally, we describe how we upscaled the pore-scale 3D geometry to a 1D model and to the geometry of a 3D shell.

### Model equations

Fgf8a gradient formation is explained by an SDD mechanism with HSPG binding (Yu et al., 2009; Harish et al., 2023). Specifically, two diffusing fractions of Fgf8a with different diffusion coefficients have been identified *in vivo*: a major fraction of ≈93% moving freely with *D*=55 μm^2^ s^−1^ and a HSPG-bound fraction of ≈7% moving with *D*=4 μm^2^ s^−1^ (Harish et al., 2023). In addition to interacting with extracellular HSPG, Fgf8a binds to cell-surface HSPG, which mediates its receptor complex formation (Schlessinger et al., 2000; Ornitz and Itoh, 2015; Gupta et al., 2024). The cell-surface bound Fgf8a fraction is not detected by single-molecule fluorescence correlation spectroscopy (FCS) measurements in the ECS but by Heparinase (HepI) injection experiments, in which cleavage of the HS side chains has been shown to lead to an increase in overall Fgf8a concentrations detected by FCS (Harish et al., 2023).

Based on these experimental findings, we account for the different Fgf8a interactions by distinguishing four concentration fields in space and time: freely diffusing Fgf8a ([Fgf8a]), slowly diffusing HSPG-bound Fgf8a in the ECS ([Fgf8a:HSPG^ECS^]), immobile cell-surface HSPG-bound Fgf8a ([Fgf8a:HSPG^cellSurf^]), and the immobile ternary 2:2:2 complex of Fgf8a, Fgfr and HSPG at the cell surface 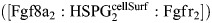. The biochemical kinetics between these species in the ECS is:
(2)

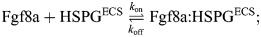
at the cell surfaces, it is:
(3)

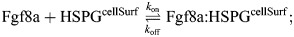
and ternary receptor complex-formation occurs as:
(4)

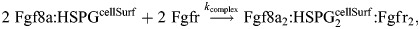
where *k*_on_ and *k*_off_ are the association and dissociation rates of Fgf8a to HSPG, and *k*_complex_ is the association rate of the ternary 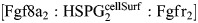 complex. As cell-surface HSPGs have been shown to trap Fgf8a and mediate receptor complex-formation and dimerization (Ornitz and Itoh, 2015; Gupta et al., 2024), our model assumes that Fgf8a binding to HSPG^cellSurf^ precedes complex formation. Furthermore, the model combines complex association, dissociation and dimerization into a single effective forward reaction to reduce the number of unknown rate constants.

Applying the principle of mass-action and assuming diffusive transport, these kinetics yield the following system of coupled partial and ordinary differential equations for the evolution of the four concentration fields in space ***x*** and time *t*:
(5)

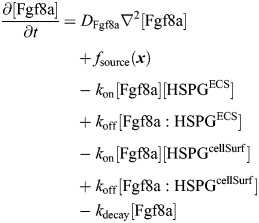

(6)

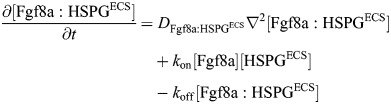

(7)

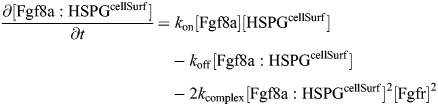

(8)

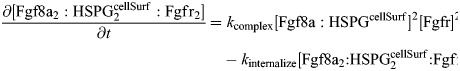
Square brackets represent concentration fields in space ***x*** and time *t*. *D*_Fgf8a_ and 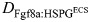 are the diffusion coefficients of the free and HSPG-bound Fgfa8 fractions in the ECS, respectively. Both diffusion coefficients are constant in space and time, neglecting spatial variations of extracellular matrix viscosity. [HSPG^ECS^] and [HSPG^cellSurf^] are the HSPG concentrations with free binding sites in the ECS and at the cell surfaces, respectively. The rate *k*_decay_ describes the decay of free Fgf8a in the ECS through thermal denaturation and proteolysis (Saksela et al., 1988; Kim et al., 2012). The rate *k*_internalize_ is the complex internalization rate through endocytosis. The model assumes that for each internalized complex, two new Fgfr and HSPG^cellSurf^ appear at the cell membrane, respectively, i.e. that their total concentration is constant over time. This simplifies the more complicated real receptor fate, of which Fgfr4 has been shown to be mainly recycled, whereas Fgfr1 with ligand is mainly degraded in the lysosome (Haugsten et al., 2005). Fgf8a secretion by source cells is represented by the source term *f*_source_(***x***).

Fgf8a sources and sinks vary in space. Concretely, only the deep cells participate in Fgf8a production and uptake (Yu et al., 2009; Stapornwongkul and Vincent, 2021), i.e. there is no production and uptake in the YSL and EVL (see Fig. 1B). We denote ECS boundaries that belong to either the EVL or YSL by ∂Ω_shell_, such that the surfaces of the deep cells are defined by 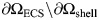. Within the deep cells, we further spatially restrict the source to a band of cells near the blastoderm margin. We define the width of this cell band *w*_source_ and its distance from the margin *d*, so that we can mathematically formalize the source term as
(9)

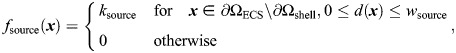
with source rate *k*_source_.

In addition to Fgf8a molecular degradation with rate *k*_decay_ everywhere in the ECS, ternary complex formation and subsequent endocytic uptake constitutes an additional Fgf8a sink *f*_sink_(***x***). This endocytic sink is localized only to the deep cell surfaces 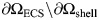 by setting it to zero everywhere else. We model *f*_sink_(***x***) as a first-order reaction with rate *k*_internalize_ as follows:
(10)

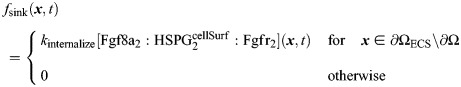


### Numerical method

To numerically model localized sources and sinks and solve Eqns 5-8 in pore-scale 3D ECS geometries derived from images, we use an open-source image-based simulation pipeline for reaction-diffusion processes in porous media (Stark and Sbalzarini, 2023). This pipeline computes a level-set representation of the diffusion domain based on volumetric microscopy images or microcomputed tomography scans. Based on the level-set function, it generates geometry-adapted sparse grids on which it solves inhomogeneous reaction-diffusion partial differential equations with multi-GPU acceleration. A detailed explanation of the numerical method has been given by Stark and Sbalzarini (2023).

The pipeline addresses the high memory requirements of multi-scale models using geometry-adapted sparse grids (Incardona et al., 2021), enabling individual point allocation in any domain subspace as needed. As sparse grids preserve the Cartesian neighborhood of a structured grid, they enable fast neighborhood access and differential operator approximation using standard grid-based schemes. Sparse grids are, therefore, suitable for discretizing porous media geometries in a memory- and compute-efficient way.

Generating such a geometry-adapted sparse grid for the complex-shaped geometry of an embryonic ECS diffusion domain Ω requires a numerical description of its boundary ∂Ω embedded in the grid, i.e. the interface between Ω and the surrounding space *S*. We use the level-set method (Osher and Sethian, 1988; Sussman and Fatemi, 1999) to implicitly represent ∂Ω as the zero-level set of a higher-dimensional function *φ*. Specifically, we use the signed-distance function (SDF):
(11)

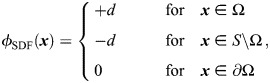
which is smooth around the surface ∂Ω and provides geometric information through the orthogonal distance *d* of any point ***x*** in the domain to the closest point on the nearest surface.

We compute *φ*_SDF_ by first generating a binary pixel mask of the ECS from the image using the pixel-classification tool ilastik (Berg et al., 2019). This represents the indicator function for Ω. Using this indicator function, we initialize *φ*(***x***, 0) with +1 inside Ω and −1 outside Ω. This serves as initial condition for computing *φ*_SDF_ using the classic Sussman level-set re-distancing algorithm (Sussman and Fatemi, 1999), which finds the SDF as the stationary solution of
(12)

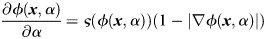
for large *α*. To prevent interface shifting across cell boundaries for steep *φ*, and to accelerate convergence of the iterations if *φ* is flat near the interface, the smoothed sign function 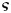 is chosen as (Peng et al., 1999):
(13)

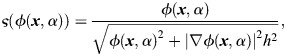
where *h* is the uniform grid spacing.

We perform Sussman redistancing with multi-CPU parallelization on a dense grid with first-order accuracy in space and time (Stark and Sbalzarini, 2023). Using the so-computed *φ*_SDF_, a geometry-adapted sparse grid containing only points in the diffusion domain is generated to reduce memory requirements. This sparse grid stores the four concentration fields along with *φ*_SDF_. Together, this enables defining spatially varying diffusion coefficients and reaction rates, including surface reactions. Using *φ*_SDF_, which indicates on which side of the surface a grid point is located, we impose zero-flux Neumann boundary conditions within the finite-difference stencil.

Taking advantage of the regular structure of sparse grids, the diffusion operators are discretized using finite-difference methods, which afford efficient memory access and parallelize well on multi-CPU and multi-GPU, in particular when an explicit time-stepping scheme is used (Jammy et al., 2019; Ye et al., 2022; Crialesi-Esposito et al., 2022). Since we here consider a strongly memory-limited problem, we solve the model in time using the first-order explicit Euler time-stepping scheme, which does not require storing and communicating intermediate stages.

### Software implementation

Even when using geometry-adapted sparse grids, the pore-scale models of the embryonic ECS exceed single-processor capacity by far, requiring efficient parallelization. We use the open-source C++ library OpenFPM for parallelizing the simulations (Incardona et al., 2019). OpenFPM data structures, which are generated at compile time using template meta-programming, hide communication from the application and are portable across CPU and GPU architectures (Incardona et al., 2023).

OpenFPM implements distributed sparse block grids by dividing the domain into chunks of 8×8×8 grid points and only allocating chunks containing at least one grid point (Incardona et al., 2021). This considerably reduces memory requirements for sparse geometries, such as the zebrafish ECS. OpenFPM sparse grids are also multi-GPU accelerated, significantly reducing simulation times compared to simulations on multi-CPU (Incardona et al., 2021). These simulations have been shown to scale with problem size and number of GPUs for irregular porous media geometries (Stark and Sbalzarini, 2023). In particular, it has been shown that the sparser the geometry and the locally denser the grid points, the larger the savings in memory and simulation run time (Incardona et al., 2021; Stark and Sbalzarini, 2023). The zebrafish ECS has a particularly sparse geometry, occupying only ≈4% of 3D light-sheet microscopy volume, making it well-suited for OpenFPM sparse-grids discretization. We visualized simulation results using Paraview (Ahrens et al., 2005). Plots were generated using the Matplotlib library (Hunter, 2007).

### Acquisition of light-sheet microscopy time-lapse video

To geometrically characterize the zebrafish ECS and its boundaries, we collected Tg(bactin:hRas-EGFP) embryos, in which enhanced green fluorescent protein (EGFP) was targeted to the cell membrane. As the embryos reached the sphere stage, they were dechorionated and injected with 0.2 nl of a 5 μm solution of tetramethylrhodamine (TMR)-labeled dextran at their animal pole, where ECS is abundant. As a hydrophilic polysaccharide, dextran does not cross the membrane barrier and distributes throughout the ECS as the embryo develops. This enabled us to track the ECS over the course of epiboly.

To perform multi-view imaging of embryos for 3D modeling, we used the Plan-Apochromat 20×/1.0W objective of the Zeiss light-sheet Z.1 microscope. For this, we first transferred the TMR-Dextran-injected embryos into a tube containing 1% low-melting point agarose, mixed with F-Z fluorescent microspheres at a 1:4000 dilution, stored at 42°C to prevent solidification. Together with the agarose-bead mix, the embryos (maximum three) were then aspirated into a glass capillary (20 μl volume, 1 mm diameter) by pulling on the inserted plunger, such that the embryos were all spaced evenly inside the capillary. Once the agarose solidified, we transferred the capillary to a beaker containing E3 medium until the imaging chamber at the microscope was assembled. Thereafter, the capillary was inserted into the imaging chamber (also filled with E3), positioned and automatically detected by imaging. An embryo embedded in the solidified agarose-bead mixture was then slowly pushed out of the capillary using the plunger until it reached the center of the detection volume.

Light-sheet adjustment, multi-view image-acquisition and later multi-view reconstruction were performed according to the protocol detailed by Icha et al. (2016) using the Fiji BigDataViewer plug-in (Preibisch et al., 2010, 2014). Image acquisition was performed from five different angles over the course of epiboly. The resulting time-lapse multi-view image data set is composed of 25 frames of 3D image volumes acquired from 5 hpf (≈40% epiboly) to 9 hpf (≈90% epiboly) (voxel size=0.4574 μm in all three dimensions, stack size: 1834×1739×1551 pixel). This allowed visualizing changes in the spatial arrangement of cells and the geometry of the ECS over the course of early development. Fig. 1C and Movie 1 show exemplary *z*-slices of the video with the EGFP-hRas-labeled cell membranes in green and the TMR-Dextran-labeled ECS in magenta (i.e. orange in Movie 1).

### Reconstructing 3D zebrafish ECS geometries based on light-sheet microscopy images

**Fig. 5.**
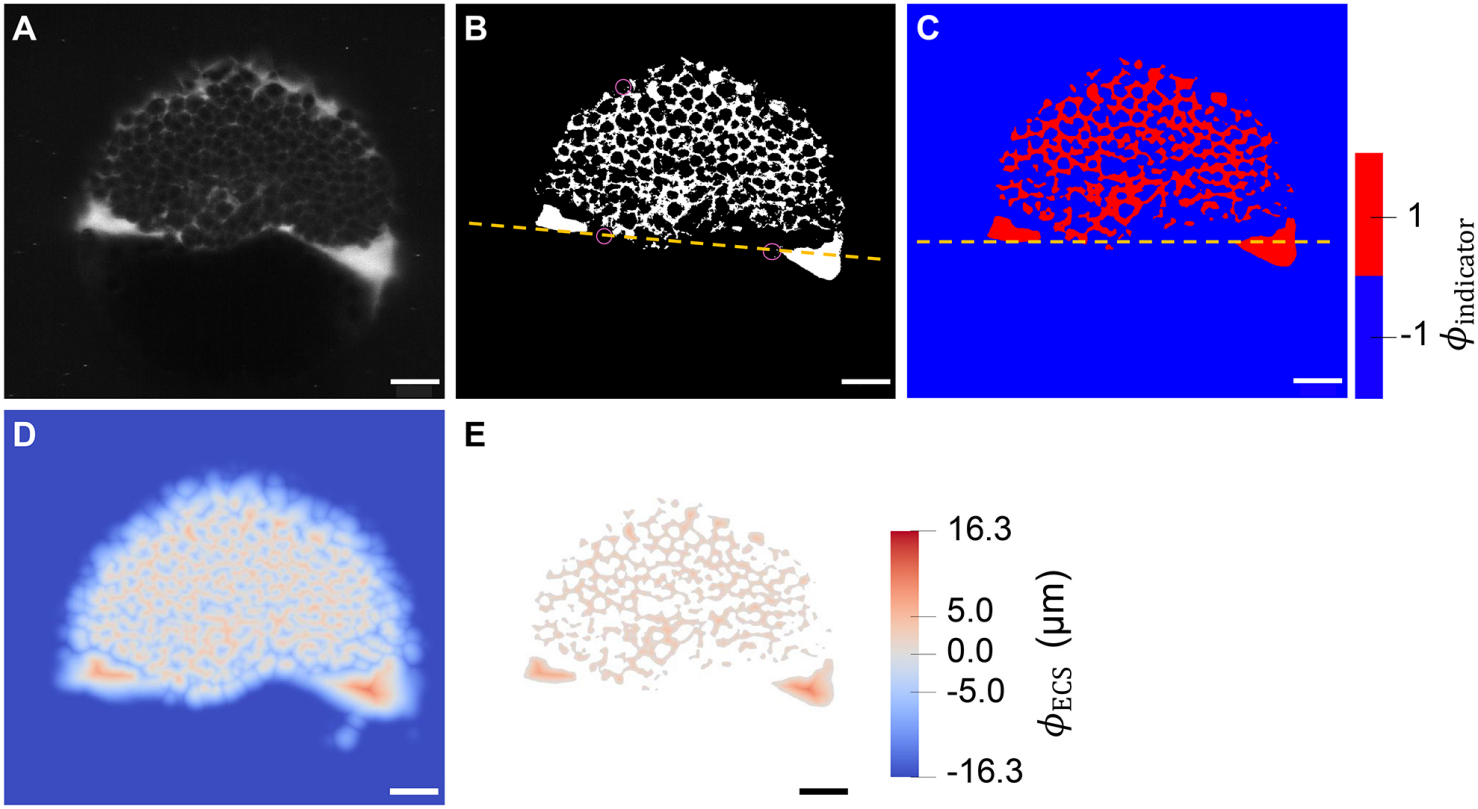
**Steps for modeling ECS geometries from light-sheet microscopy volumes.** Volumes are visualized for an exemplary time point (12 out of 25, ≈60% epiboly) and *z*-slice (plane 480 from the epiblast in the direction of hypoblast and the yolk, i.e. the mid-plane formed by the AV/DV axes). (A) TMR-dextran channel of the light-sheet data after 3D multi-view fusion; fluorescence marks the ECS. (B) Segmentation mask of the ECS (white) with a yellow dashed line showing the margin plane and pink circles highlighting specks of noise and small unconnected islands that were subsequently removed. (C) Binary indicator function of the ECS after mask denoising and horizontal alignment of the margin plane (yellow dashed line). (D) Re-distanced signed-distance function, *φ*_ECS_, of the level-set representation of the ECS surface. (E) Sparse block grid storing only points in the ECS. Scale bars: 50 μm.

Using the TMR-dextran fluorescence signal of the light-sheet time-lapse video, we modeled ECS geometries using level-set sparse grids, as explained in the sections ‘Software implementation’ and ‘Numerical method’. This image-based ECS reconstruction consisted of five steps, the intermediate results of which are shown in Fig. 5A-E for an exemplary *z*-slice. The steps were: (1) extracting volumes of individual time points from the light-sheet video; (2) image processing and segmentation using Fiji (Schindelin et al., 2012) and ilastik (Berg et al., 2019); (3) processing of the segmentation result and conversion to an indicator function; (4) computing a numerical surface description as a signed distance function (SDF) using the level-set method; and (5) generating a geometry-adapted discretization of the ECS using distributed sparse block grids (Incardona et al., 2021). We next describe these five image-based modeling steps in more detail.

The light-sheet video (exemplary *z*-slices in Fig. 1C) was stored as a 0.7 TB HDF5 file containing five viewpoints plus their 3D fusion for all 25 time points. For modeling the geometry, 3D fused views were extracted as separate files to reduce RAM/DRAM requirements. Extraction of the 3D multiview reconstruction of the TMR-dextran channel at each time point was carried out using the Multiview Reconstruction Tool from the Fiji BigDataViewer plug-in (Preibisch et al., 2010, 2014). An exemplary *z*-slice is shown in Fig. 5A.

The resulting TIFF files were subsequently converted to the ilastik HDF5 file format using the ilastik Import/Export plugin in Fiji. In ilastik (Berg et al., 2019), the interactive Random Forest pixel classifier was used to obtain a pixel mask for the ECS. The classifier was trained by manual image example annotation for the two pixel classes, ECS versus non-ECS, where non-ECS included pixels of background, cells and yolk. Pixel class annotation and prediction were repeated for different *x*, *y* and *z*-slices of the 3D image volume at video time point 12 until the classification results no longer improved. The trained classifier was then used for pixel classification of the other 24 time points of the video without further manual annotation.

The segmentation result, shown in Fig. 5B for an exemplary *z*-slice, likely overestimates the ECS volume because ECS structures can be below pixel resolution. Nevertheless, the network character of the ECS geometry was successfully captured.

Postprocessing of the segmentation mask (pixel value of 2 in the ECS and 1 otherwise) was carried out before conversion to an indicator function. This included Gaussian filtering (*σ*=2) and alignment of the sample rotation using the scipy.ndimage Python package (Virtanen et al., 2020). Sample alignment made the margin plane horizontal (yellow dashed line in Fig. 5B,C) by rotation of the 3D pixel mask about the *z*-axis, applying the standard rotation matrix using trigonometric functions. The rotated mask was then interpolated back to the pixel grid using third-order spline interpolation. This was followed by thresholding the filtered and rotated segmentation mask, which was no longer binary, setting pixels to one with value >1.4 and all others to −1. The threshold was determined such that it removes unconnected islands of size ≤3×3×3 pixels, whose maximum value after Gaussian smoothing was 1.39 (examples marked by pink circles in Fig. 5B). This denoising step was necessary to avoid numerical instabilities in the Sussman algorithm that would otherwise be caused by imaging noise, at the expense of introducing a small bias and artificial smoothing of the ECS surface. This resulted in an indicator field, an example of which is shown in Fig. 5C, that was stored as a binary file. Each of these binary files was 162.5 GB in size. The indicator function was then loaded onto a distributed-memory 3D computational grid on a compute cluster with multiple CPUs. Thin structures <2 pixel in width were removed to fulfill the level-set resolution requirement resulting from the Nyquist-Shannon sampling theorem (Stark and Sbalzarini, 2023).

Finally, a representation of the ECS surfaces was computed as a SDF *φ*_ECS_, as explained in the section ‘Numerical method’. The exemplary result is shown in Fig. 5D. Based on this *φ*_ECS_, a sparse block-grid representation of the ECS was generated, resulting in a memory-efficient geometry-adapted data structure, as illustrated in Fig. 5E. A full 3D visualization of the resulting surface is shown in Fig. 1D-G for the time point at ≈60% epiboly. Following this procedure, we built models of the ECS geometries for all 25 time points of the light-sheet time-lapse video. Visualizations of the results at all time points are shown in Fig. S10.

At full resolution, with one grid point per light-sheet microscopy voxel, representing the zebrafish ECS on a sparse block grid reduced memory requirements by a factor of 18, from ≈306 GB (dense grid, no blocks) to ≈17 GB (sparse block grid). This enables fully resolved numerical simulations on a GPU, since the sparse-grid data fits the DRAM of a typical GPU (here: Nvidia A100 with 40 GB DRAM). We further found that simulation results at full and half grid resolution were identical, indicating that the pixel resolution of the geometry was sufficient to yield grid-converged numerical results. Using half the resolution resulted in an overall 28-fold reduction in simulation times (from 110 h to 4 h of GPU compute time per run simulating 60 min of physical time). We therefore used a half-resolution computational grid (i.e. *h*=2pixel) in all simulations, resulting in a grid spacing of 0.915 μm and a memory requirement of 3.5 GB.

As the time resolution of the light-sheet microscopy video was not fine enough to allow the derivation of continuous ECS deformation maps, we simulated gradient formation in fixed ECS geometries, neglecting advective transport and growth. We imposed zero-flux Neumann boundary conditions at all interfaces, assuming that the fluid inside the ECS does not permeate the cell membranes. With these assumptions and using the numerical methods from the section ‘Numerical method’, we solved the model equations from the section ‘Model equations’ in the 3D ECS geometries at ≈40%, 60% and 75% epiboly to study morphogen gradient emergence and maintenance.

### Modeling localized sources and sinks

In order to spatially restrict sources and sinks according to Eqns 9 and 10 to the surfaces of the deep cells, these surfaces need to be distinguished from those of the YSL and EVL, i.e. what we call the shell boundary – ∂Ω_shell_. To accomplish this, we computed another SDF, *φ*_shell_, representing ∂Ω_shell_, following the procedure described in the section ‘Reconstructing 3D zebrafish ECS geometries based on light-sheet microscopy images’, but using segmentation masks in which the cells have been blurred out by Gaussian filtering (*σ*=3) followed by setting pixels to one whose value >1.0 and all others to −1.

**Fig. 6.**
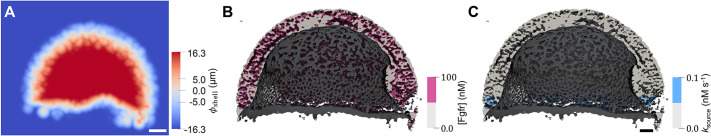
**Spatially varying sources and sinks in the 3D model at an exemplary time point at ≈60% epiboly.** (A) Signed-distance function (SDF) of the embryo shell boundary, *φ*_shell_, for an exemplary *z*-slice. Combining *φ*_shell_ (representing YSL and EVL boundaries) with *φ*_ECS_ enables the restriction of sources and sinks to the deep-cell surfaces. (B,C) A clipped model of the ECS showing that Fgf receptors (B, pink) are restricted to the deep cell boundaries, i.e. not the EVL or YSL. The sources (C, blue) are restricted to ≈5 rows of deep cells above the margin of the blastoderm. Receptor concentrations are given in units of nM; *k*_source_ is given in nM/s. Scale bars: 50 μm.

This resulted in a SDF as shown in Fig. 6A. Combining *φ*_shell_ and *φ*_ECS_ enables defining the deep-cell surfaces as the set of grid points 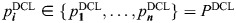 within one grid spacing from ∂Ω_ECS_ and further than 15 μm away from ∂Ω_shell_, i.e. 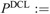 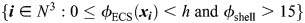, to which we restrict [Fgfr] and [HSPG^cellSurf^].

Fgf8a secretion was also restricted to this same set of grid points, additionally restricting the source location to a band of deep cells above the blastoderm margin (Scholpp and Brand, 2004). The width of this band was ≈5 cells at early gastrula (Harish et al., 2023) and ≈8 cells at mid gastrula (Scholpp and Brand, 2004). For this, *w*_source_ in Eqn 9 was set to 70 μm at 40% and 60% epiboly, and to 112 μm at 70% epiboly, assuming an average cell diameter of 14 μm (Harish et al., 2023). The model assumes that source cells also act as sink cells, in accordance with *in vivo* observations of Fgfr expression (Ries et al., 2009; Ota et al., 2010). Comparing the simulation results of this model with a model that assumes source cells do not act as sinks, we found that the normalized gradient profiles are unaffected by this assumption (see Fig. S2). The resulting sources and sinks of the model are visualized in Fig. 6 for an exemplary time point at ≈60% epiboly.

### Parameter search

A major difficulty of modeling morphogen gradient formation realistically, such that it can be validated with experimental data, is that various required key parameters remain unknown because they are difficult or impossible to measure experimentally. These include morphogen production and degradation rates, receptor and non-receptor binding affinities and concentrations, and advection velocities due to growth. The models hence remain underdetermined with multiple unknown parameters, requiring parameter searches and fitting (Solomatina et al., 2022).

The model described in Eqns 5-8 has nine unknown parameters, in particular, [Fgfr], [HSPG^ECS^], [HSPG^cellSurf^], *k*_on_, *k*_off_, *k*_decay_, *k*_complex_, *k*_internalize_ and *f*_source_. We performed a systematic parameter search to find values of the parameters that maintain the *in vivo* gradient.

To maximize the predictive power of our model, we fixed as many parameters as possible to experimental or literature values, as summarized in Table 2. One of the unknown parameters is the overall binding and unbinding rate of Fgf8a to HSPG^ECS^ and HSPG^cellSurf^. For the two HSPG families, glypicans and syndecans, the dissociation constants, defined as 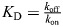, of the Fgf8-HSPG interaction 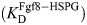 have been determined by *in vivo* dual-color fluorescence cross-correlation spectroscopy (FCCS) in the gastrulating zebrafish: *K*_D_=1.03…1.24 μM (syndecans) and *K*_D_=6.15±0.74 μm (glypicans) (Gupta et al., 2024). Hence, both affinities are more than an order of magnitude lower than Fgf8-receptor binding affinities (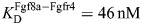 and 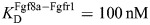; Ries et al., 2009).

**Table 2.** Summary of all model parameters

Model parameter	Symbol	Value	Source*
Diffusion coefficients			
Fgf8a (93% of extracellular Fgf8a)	*D*_Fgf8a_	55 μm^2^ s^−1^	Harish et al., 2023
Fgf8a:HSPG (7% of extracellular Fgf8a)	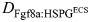	4 μm^2^ s^−1^	Harish et al., 2023
Concentrations			
Maximal concentration of extracellular Fgf8a		7.9 nM	Exp.
Extracellular HSPG concentration	[HSPG^ECS^]	0.4 μM	Search
Cell-surface HSPG concentration	[HSPG^cellSurf^]	1.0 μM	Search
Fgf receptor concentration	[Fgfr]	0.1 μM	Text
On and off binding rates			
Association rate of Fgf8a and HSPG^ECS/cellSurf^	*k*_on_	4.6×10^−6^ (nM s)^−1^	Text
Dissociation rate of Fgf8a and 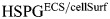	*k*_off_	0.023 s^−1^	Sperinde and Nugent, 2000; Filion and Popel, 2004
Dissociation constant of Fgf8a:HSPG^ECS/cellSurf^	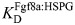	5.0 μM	Gupta et al., 2024; Text
Association rate of Fgf8a:HSPG^cellSurf^ and Fgfr Source and sink rates	*k*_complex_	4.6×10^−5^ (nM s)^−1^	Text
Fgf8a production rate	*k*_source_	0.1 nM s ^−1^	Search
Fgf8a molecular decay rate	*k*_decay_	1.3×10^−3^ s^−1^	Yu et al., 2009
Internalization rate for 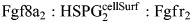	*k*_internalize_	9×10^−3^ s^−1^	Text

*We combined literature values (references) with own experimental measurements (Exp.) and deductive reasoning from known values as described in the text (Text). The remaining three parameters were determined in a computational parameter search (Search).

Assuming that the syndecans and glypicans affinities are representative of the overall Fgf8a:HSPG interaction, and considering that more glypicans than syndecans are present in the zebrafish ECS (Gupta et al., 2024), we set the dissociation constant of Fgf8a:HSPG^ECS^ and Fgf8a:HSPG^cellSurf^ in our model to an average 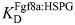 of 5.0 μm. Using this 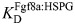 and an HSPG unbinding rate *k*_off_=0.023 s^−1^, as has been measured for Fgf2 (Sperinde and Nugent, 2000; Filion and Popel, 2004), we find the binding rate 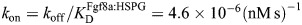. Furthermore, we assume a 10-times higher Fgfr binding rate of *k*_complex_=4.6×10^−5^(nM s)^−1^ and set [Fgfr]=0.1 μm by approximating volume concentrations from experimentally determined Fgfr surface concentrations (Ries et al., 2009).

It has been experimentally shown that Fgf8a concentrations are higher at the membrane than in the ECS (Gupta et al., 2024) and that cleavage of HS side-chains upon heparinase injection causes a two-fold increase in Fgf8a concentration levels in the ECS (Harish et al., 2023). The exact factor by which [HSPG^cellSurf^] is higher than [HSPG^ECS^] is, however, unknown, so we set it arbitrarily to 2×.

The decay length of the steady-state gradient for non-recycled morphogens depends on the effective degradation rate *k*_degradation_ and the effective diffusion coefficient *D*_eff_ as: 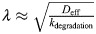 (Kicheva et al., 2012; Gordon et al., 2013; Hidalgo et al., 2019 preprint). Taking into account geometrical hindrance, as discussed in the section ‘Determining the ECS diffusive tortuosity to quantify geometric hindrance’ and the gradient decay length *λ* from the *in vivo* gradient profile in Fig. 14A, we estimate
(14)

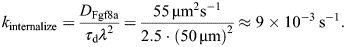
This estimation of *k*_internalize_ is only an initial guess, as it does not consider the rate of complex formation, which is required for internalization, and the molecular decay in the ECS. It is, however, of the same order of magnitude as the experimentally measured *k*_decay_=1.3×10^−3^ s^−1^ (Yu et al., 2009). We further found in the sensitivity analysis that the normalized gradient is not sensitive to the choice of *k*_internalize_ (see Fig. 3B).

**Fig. 7.**
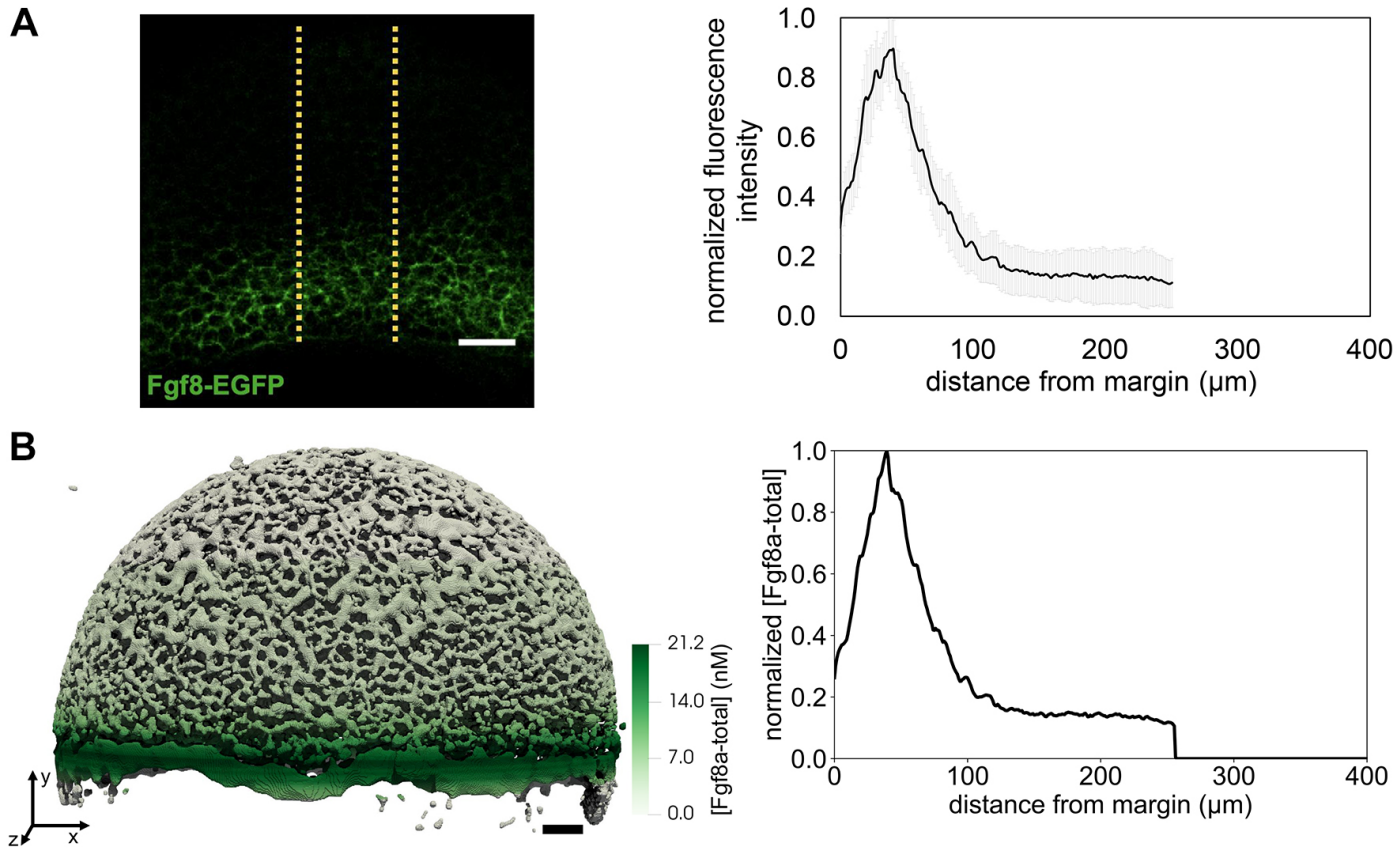
**Initialization of the total Fgf8a concentration with the experimental fluorescence intensity profile at ≈60% epiboly.** (A) The fluorescence intensity profile (right) is extracted from sum-intensity *z*-projected confocal images (left). Values are intensity averages across a 100 μm wide region (neural plate). Reproduced, with permission, from Harish et al. (2023). The embryo is oriented as in Fig. 1B (ventral left, dorsal right, animal pole top, vegetal pole bottom). *N*=15 embryos. Data are represented as mean±s.d. (B) The concentration field in the simulation (left) was obtained by linearly interpolating between the discrete margin distances of the experimental profile and uniformly distributing the mass in each *xz*-plane. We converted normalized intensity values to concentrations by scaling with the maximum concentration measured by FCS. For comparison with experiments, we computed 1D profiles (right) along the AV axis by averaging the total Fgf8a concentration in each *xz*-plane. The model has the same orientation as the embryo in A. Scale bars: 50 μm.

For the computational parameter search, we assumed that the experimentally measured intensity profile at 60% epiboly was at a steady state for this time point. We then searched for values of the remaining three parameters (‘Search’ in Table 2) that maintained this steady-state profile. For this, we initialized the Fgf8a concentration in the geometry reconstruction at 60% epiboly with the experimental profile shown in Fig. 7A, as detailed in the section ‘Grid initialization with experimental gradient’. To compare the 3D simulation results with the 1D experimental gradient, we computed 1D profiles along the AV axis by averaging the sum of all four Fgf8a fractions in each *xz*-plane of the simulation grid. These concentration averages were subsequently normalized and plotted against the distance from the blastoderm margin, as shown in Fig. 7B. Using the experimental profile as initial condition, we computed the solution of Eqns 5-8 for different parameter sets until 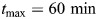, which was sufficiently long to ensure that gradients have reached a steady state.

**Fig. 8.**
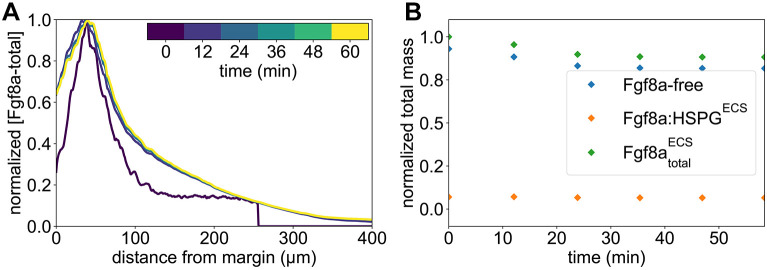
**Simulated Fgf8a gradient using optimized parameters with experimental profile as initial condition.** For parameters, see Table 2. (A) Normalized AV concentration profiles of Fgf8a-total at different simulated times (see color intensity bar). All profiles are normalized by their respective maximum *xz*-plane-averaged concentration. The experimental profile used as the initial condition is shown in dark purple (time 0). Simulated profiles reach a steady state within 12 min. (B) Normalized total mass of two Fgf8a fractions, Fgf8a:HSPG^ECS^ and Fgf8a-free, along with their sum (Fgf8a-total). This set of parameters maintains a constant total Fgf8a mass and preserves the *in vivo* Fgf8a/Fgf8a:HSPG^ECS^ ratio of 93%/7%.

With these assumptions, and using the experimental profile as initial condition, we tested different *k*_source_ and HSPG concentrations in simulations. These two parameters mainly influenced the Fgf8a/Fgf8a:HSPG^ECS^ ratio in the simulations, i.e. the higher they were, the higher the bound fraction. We found that [HSPG^ECS^]=0.4 μm and [HSPG^cellSurf^]=1.0 μm closely matched the experimentally measured Fgf8a/Fgf8a:HSPG^ECS^ ratio (simulated ratio: 92.6%/7.4%) after 60 min of simulated time (see Fig. 8B). For these parameter values, the concentration levels of the overall Fgf8a profile were well maintained when using a source rate of 0.1 nM s^−1^, as shown in Fig. 8A.

Although maintaining the Fgf8a/Fgf8a:HSPG^ECS^ ratio and the overall profile, the simulated profile converges within 12 min to a steady-state profile slightly flatter than the experimental profile (Fig. 8A) and a lower peak concentration (see absolute concentration profiles in Fig. S3). Possible explanations are that some of the parameter values are not correct, the experimental input profile is not at a steady state or there is lower geometrical hindrance due to overestimated ECS connectivity and volume. These are a consequence of image-segmentation errors discussed in the section ‘Reconstructing 3D zebrafish ECS geometries based on light-sheet microscopy images’ and quantified in the section ‘Tortuous ECS geometries stabilize Fgf8a gradients’. Despite not perfectly maintaining the slope of the input profile, the simulated profile maintains a drop close to the margin, likely caused by a zonation effect in the marginal region with low cell density and poor connectivity to the rest of the ECS (compare with Fig. 7B). Furthermore, the normalized profiles maintain the overall shape of the *in vivo* gradient (Fig. 8A).

Overall, the parameters shown in Table 2 preserve the experimentally measured gradient at 60% epiboly and reproduce the *in vivo* ratio of Fgf8a/Fgf8a:HSPG^ECS^. We use these parameters throughout the study.

### Determining the ECS diffusive tortuosity to quantify geometric hindrance

**Fig. 9.**
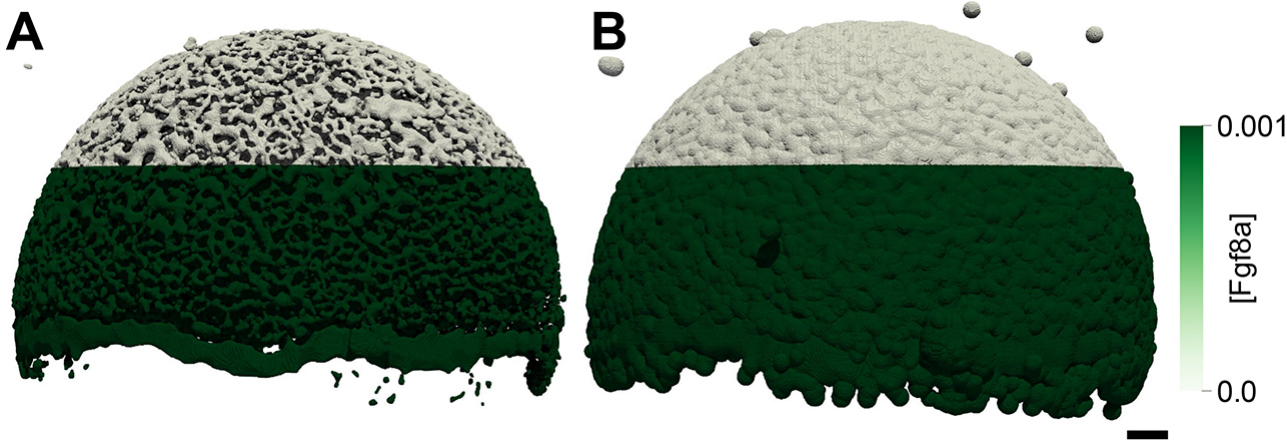
**Initial condition of simulated FRAP experiment in the ECS geometry and the shell geometry.** (A) ECS geometry and (B) shell geometry. The lower half of the ECS is uniformly initialized with [Fgf8a]=0.001 (arbitrary unit), and the upper half (above 500 μm above the margin) with [Fgf8a]=0. To estimate the diffusive hindrance of ECS tortuosity alone, we simulate diffusion without reactions, measuring the total mass recovery in the bleached half. Fitting the recovery curve of the shell geometry to the curve of the ECS geometry, we find the ratio between the local and effective diffusion coefficient, i.e. the tortuosity factor *τ*_d_=2.5. Scale bar: 50 μm.

As shown in the section ‘Tortuous ECS geometries stabilize Fgf8a gradients’, the Fgf8a AV gradient is sensitive to changes in the ECS geometry. However, the impact of the geometry may only be indirect through factors such as the total surface areas of source and sink cells, or the total volume (and hence mass) of HSPG. We quantify the diffusive hindrance from the ECS geometry alone by computing the ECS diffusive tortuosity *τ*_d_ (see Eqn 1). We determine *τ*_d_ of the 3D ECS geometry at 60% epiboly by simulating a FRAP experiment. For this, we initialize the lower half of the ECS with a uniform concentration field and set the concentration in the upper half (above 500 μm above the margin) to zero, as shown in Fig. 9A. We then simulate pure diffusion, i.e. without HSPG binding, sources and sinks, and measure the time required for the concentration field to uniformly distribute throughout the ECS. This time depends on the molecular diffusion constant *D* and on the modeled ECS geometry. Comparing this time to the one determined in a plain spherical shell (see Fig. 9B) of identical size and with identical *D*, we find *τ*_d_=2.5. This means that – excluding other hindrance factors –diffusive transport is less than half as efficient in the ECS geometry than in an empty shell without cells.

This value agrees with recent tortuosity estimations of the zebrafish brain ECS at 24 hpf, where the ECS has been reconstructed based on combined fluorescence and transmission electron microscopy images (Zhu et al., 2024). In this reconstruction, FRAP has been simulated using an agent-based particle Monte-Carlo method, and *τ*_d_ has been estimated to range from 2.0 to 2.7 for different parts of the brain (Zhu et al., 2024). Interestingly, this value is comparable to the diffusive tortuosity of circumventing a 2D disk compared to crossing the disk, i.e., 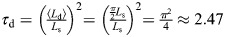 (see also Müller et al., 2013). The *τ*_d_ measured for the ECS thus seems to reflect the spherical shapes of the cells that the molecules have to circumvent.

### Upscaling 3D porous-media characteristics of the ECS

We have shown in the section ‘Fgf8a normalized gradients are robust to changes in rate constants but sensitive to changes in ECS geometry’ that Fgf8a AV gradients are robust to changes in source and sink rates when simulated in realistic ECS geometries, but are sensitive to changes in the ECS geometry, the molecular diffusion constant and transient HSPG binding. This suggests that diffusive hindrance may be a core mechanism in morphogen gradient emergence and robustness. Diffusive hindrance is a core concept of transport in porous media (Archie, 1942; Sbalzarini et al., 2005; Ghanbarian et al., 2013; Etancelin et al., 2020). In the present system, diffusive hindrance results from the joint effect of geometric tortuosity, transient HSPG binding in the ECS and at the cell surfaces, receptor complex formation, and the finite molecular diffusion constant of the morphogen. In this sense, the ECS geometry, with its extracellular HSPG, effectively acts as a porous medium regulating Fgf8a gradient shape.

**Fig. 10.**
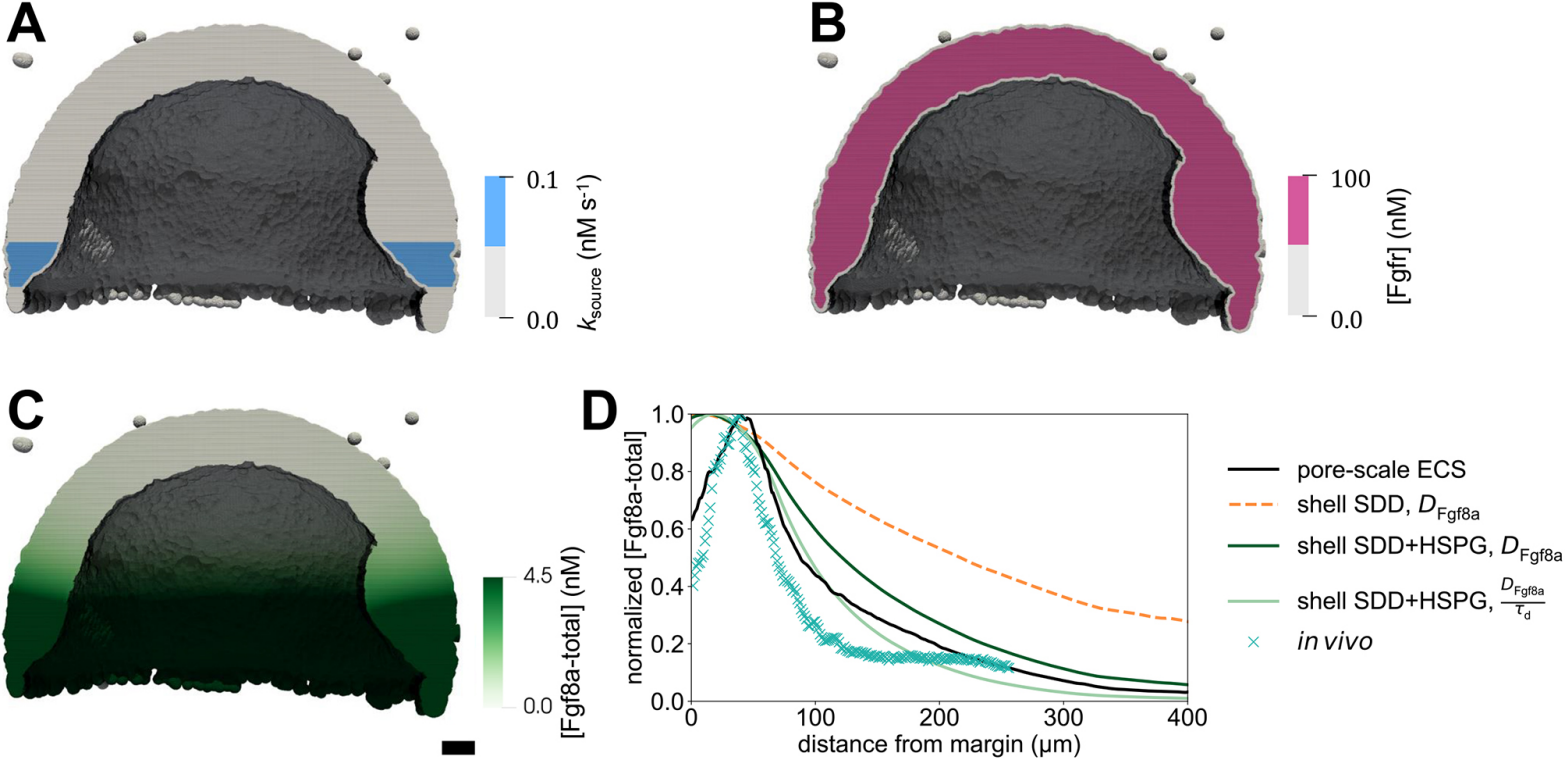
**Porous-media characteristics of ECS can be partly upscaled in a shell geometry.** (A-C) Clipped simulation visualization of (A) the source in a 70 μM DCL band at the margin, (B) the uniformly distributed sink ([Fgfr]) in the DCL, and (C) the Fgf8a gradient at 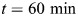 (concentration color bar) using the parameters from Table 2. (D) Modeling the ECS geometry as a spherical shell without HSPG leads to a longer, flatter gradient (orange dashed line) compared to the baseline of the pore-scale model (black line). Gradient steepness and range are partly recovered by upscaling HSPG binding by uniformly distributed HSPG^ECS^ and HSPG^cellSurf^, and they are fully recovered when further upscaling the tortuosity by an effective diffusion coefficient using *τ*_d_ (as defined in the section ‘Determining the ECS diffusive tortuosity to quantify geometric hindrance’). However, this does not apply to local geometry effects such as the zonation near the source, as found in the *in vivo* profile (indicated by cyan crosses) and reproduced by the pore-scale model. AV profiles are reported at 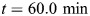. Scale bar: 50 μm.

Our model resolves the porous medium explicitly by resolving geometry and binding reactions at the ‘pore scale’. To test whether Fgf8a gradient shape can be explained by an effective porous medium, we compare simulations in a fully resolved ECS geometry with simulations in an upscaled ECS. In this upscaling, the geometry is simplified to a spherical shell by extracting the outer surfaces of our ECS geometry model at 60% epiboly, filled with a uniform porous material (see Fig. 10). This porous material models the coarse-grained effect of ECS geometry and diffusive hindrance without explicitly resolving them. In this simplified model, surface reactions and diffusive tortuosity are represented by uniformly distributed effective volume rates. The source is uniformly distributed within a deep-cell band at the blastoderm margin with the same width as in the fully resolved geometry model (70 μM), as shown in Fig. 10A. Receptor-mediated endocytosis is modeled throughout the DCL, as shown in Fig. 10B.

We first quantify the effect of HSPG binding by simulating Fgf8a gradient formation by an SDD mechanism without HSPG binding and Fgf8a degradation at rate *k*_decay_ in the entire shell. Using this upscaled model, we simulate *de novo* gradient formation with the parameters given in Table 2 until a steady state is reached at 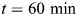. The normalized gradient profiles in Fig. 10D show that the gradient in the spherical shell geometry emerging from an SDD mechanism without HSPG (orange dashed line) does not reproduce the baseline gradient (black line). The gradient in the spherical shell is shallower with a longer propagation length and lacks the zonation near the margin. The latter is because in the coarse-grained model, the marginal region is fused with the shell, whereas the real geometry has a porosity gradient along the AV axis, as discussed in the section ‘Computing porosity profiles along the AV and DV axes’. The flatness and long range can be explained by missing diffusive hindrance from HSPG and Fgfr binding, and ECS tortuosity.

To upscale diffusive hindrance by HSPG binding and complex formation, we assume uniformly distributed HSPG^ECS^ and HSPG^cellSurf^ in the entire shell and receptor-mediated endocytosis throughout the DCL (see Fig. 10B). The diffusive hindrance provided by HSPG binding and receptor-complex formation in the entire volume of this geometry is stronger than in the baseline ECS geometry, where these effects are restricted to the cell surfaces. Fig. 10D shows that, under these modeling assumptions, *de novo* formed gradients are steeper and shorter (dark-green line) than with an SDD mechanism alone. Although this gradient is still shallower than the baseline and lacks the zonation near the source, gradient steepness is partly recovered by this upscaling of HSPG binding.

In addition to HSPG binding, the effect of geometric hindrance can be upscaled using the diffusive tortuosity *τ*_d_=2.5 of the ECS geometry, as defined in Eqn 1 and determined in the section ‘Determining the ECS diffusive tortuosity to quantify geometric hindrance’. This factor describes how strongly the ECS geometry alone hinders Fgf8a diffusion. Limited by the light diffraction limit and segmentation errors, our geometry model likely overestimated the thickness of ECS structures due to light. We, therefore, expect the real *τ*_d_ to be even larger (Zhu et al., 2024).

Simulating gradient formation in the spherical shell with upscaled diffusion coefficient 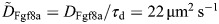 indeed recovers gradient steepness and range (see Fig. 10D, light-green line). The geometric zonation effect near the source, however, is still not reproduced, as this local effect is not captured by the averaged tissue-wide factor.

In summary, these results show that the ECS effectively acts as a porous medium when modulating morphogen gradient shape. In this, geometric tortuosity acts in concert with matrix and receptor binding to create the required diffusive hindrance. Hindrance from matrix binding alone does not account for the correct gradient slope. The effective geometric hindrance, as represented by the upscaled tortuosity, restores the global gradient shape but not the local zonation effects. This shows that the porous-media characteristics of the ECS geometry are sufficient to explain morphogen gradient shape.

### Computing porosity profiles along the AV and DV axes

**Fig. 11.**
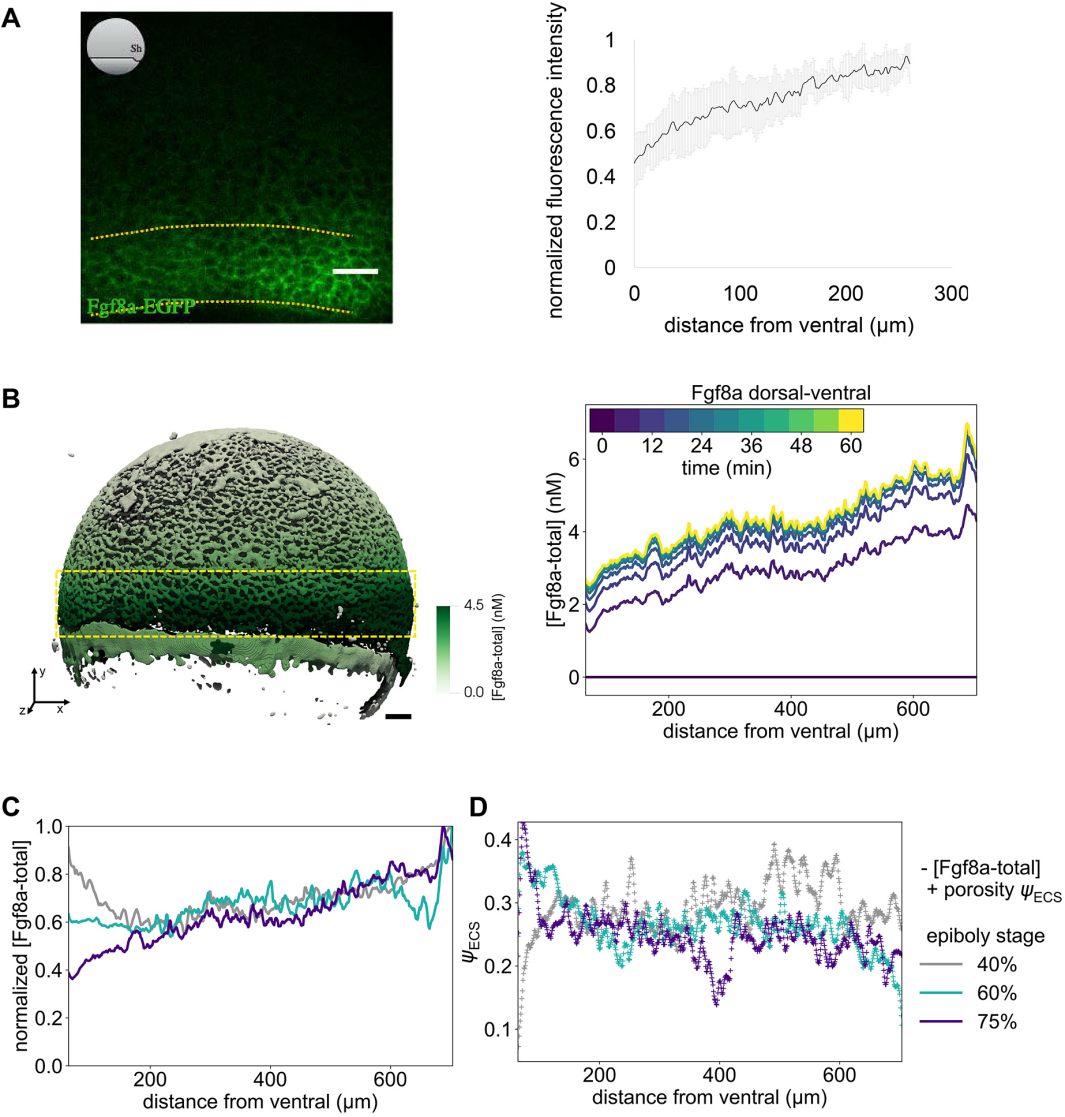
**Asymmetry in ECS geometry could explain Fgf8a DV gradient.** (A) The Fgf8-EGFP gradient in the ECS at midgastrula stage (≈75% epiboly). The normalized fluorescence intensity profile in the ventral-to-dorsal direction (right) was extracted from sum-intensity *z*-projected confocal images (left). Values are intensity averages across a region at the marginal blastomere (dotted yellow lines); *N*=10 embryos. Data are mean±s.d. The embryo is oriented as in Fig. 1A (ventral left, dorsal right, animal pole top, vegetal pole bottom; see gray schematic). Reproduced, with permission, from Harish et al. (2023). (B) Simulated *de novo* Fgf8a gradient and DV concentration profiles at ≈75% epiboly. The concentration profiles in the ventral-to-dorsal direction (right) were computed by averaging concentration values over *yz*-planes within the marginal region (dashed yellow lines). The model has the same orientation as the embryo in A. Color represents concentrations in nM (see intensity bar on the left) and simulated time of gradient formation (see color intensity bar on the right). Scale bars: 50 μm. (C) Simulated normalized *de novo* Fgf8a DV concentration profiles at different stages of epiboly (see key). A gradient similar to the one observed *in vivo* (A) emerges at ≈75% epiboly. The computationally predicted profiles are flat in geometries of 40% and 60% epiboly (gray and cyan lines, respectively). All profiles are computed at 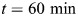. (D) ECS porosity (plus symbols) along the DV axis at different stages of epiboly (see key). At 75% epiboly, the *ψ*_ECS_ profile shows a gradient with porosity values approximately halving from ≈0.4 at the ventral to ≈0.2 at the dorsal side. The hypothesis that this could explain the Fgf8a DV gradient is contradicted by the similar *ψ*_ECS_ profile at 60% epiboly, which is when the Fgf8a DV profile is flat.

The tortuosity determined in the section ‘Determining the ECS diffusive tortuosity to quantify geometric hindrance’ and used for upscaling in the section ‘Upscaling 3D porous-media characteristics of the ECS’ is an average value that does not capture anisotropies and heterogeneities of the ECS. Therefore, it cannot be used to study the effects of geometric asymmetries on gradient formation. We next aimed to elucidate the role of ECS asymmetries in shaping the Fgf8a gradient. In particular, it has been observed *in vivo* that, in addition to the gradient along the AV axis, Fgf8a forms a weaker gradient along the DV axis within the marginal blastomere with increasing Fgf8a concentration towards the dorsal side, as shown in Fig. 11A at ≈75% epiboly (Harish et al., 2023). This gradient could be caused by spatial variations in source or sink rates, or it could be a consequence of DV asymmetry of the ECS geometry with a thicker shield cell layer at the dorsal side.

To test whether geometric asymmetry is sufficient to explain the DV gradient, we compute *de novo* 1D concentration profiles along the DV axis for different time points at ≈75% epiboly by averaging concentrations over *yz*-planes in a region 100 μm above the margin (dashed yellow lines in Fig. 11B, left). The resulting concentration profiles (Fig. 11B, right) show that a gradient comparable to the experimentally observed gradient (Fig. 11A, right) emerges over time and reaches steady state after ≈30 min. Since all other model parameters, in particular all sink and source rates, were kept constant along the DV axis, this shows that asymmetries in the ECS geometry are sufficient to explain the Fgf8a DV gradient. This is in line with the observation that DV concentration profiles are flat in ECS geometries of earlier epiboly stages, where geometric asymmetries are not yet as pronounced (Fig. 11C).

While this shows that geometric asymmetries are sufficient, they may not be necessary. Additional factors may also contribute to the DV gradient, such as spatial asymmetries in source rates. For example, it has been shown *in vivo* that Fgf8a-mRNA concentrations are higher in the cells at the dorsal side (Fürthauer et al., 1997; Reifers et al., 1998). Another factor could be faster growth (and thus advection) rates at the dorsal side, as it has been shown that, beyond 50% epiboly, cells at the dorsal side move faster than cells at the ventral side (Li et al., 2022).

**Fig. 12.**
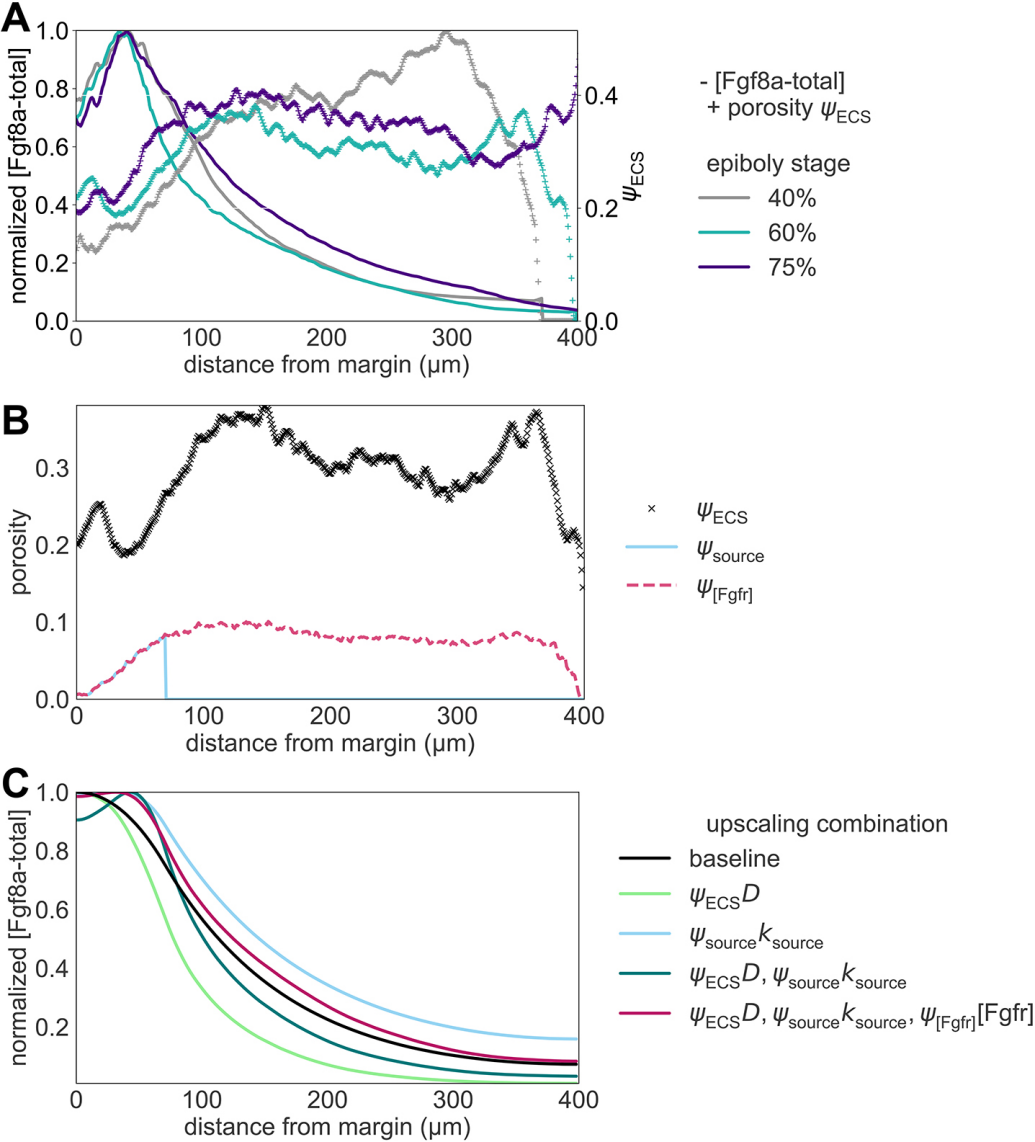
**Using porosity profiles along the AV axis for upscaling in a 1D model.** (A) Comparison of the ECS porosity (plus symbols, right *y*-axis) and [Fgf8a-total] profiles (lines, left *y*-axis) along the AV axis at different stages of epiboly (see key). In the first 100 μm from the margin, where the profile has a kink, *ψ*_ECS_ approximately doubles. All profiles are computed at 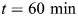. (B) AV profiles of the ECS porosity, *ψ*_ECS_ (black symbols); the source, *ψ*_source_ (solid light-blue line); and the sink, *ψ*_[Fgfr]_ (dashed pink line). All profiles are computed according to Eqn 15 by dividing the volume of the respective phase of interest by the total volume of the embedding spherical shell. (C) Upscaling SDD in porous ECS geometries. To disentangle the role of porous ECS heterogeneity in source, diffusion and sink on the formation of the AV gradient kink, we accounted for the AV profiles of *ψ*_ECS_, *ψ*_source_ and *ψ*_[Fgfr]_ by linearly scaling (1) *D*_Fgf8a_ and 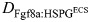 with *ψ*_ECS_ (light-green line) and (2) *f*_source_ with *ψ*_source_ (light-blue line), and by (3) combining (1) and (2) (dark-green line), and (4) combining (3) with scaling [Fgfr] with *ψ*_[Fgfr]_ (pink line). The kink at the source is partly recovered when upscaling *D*_Fgf8a_ and *f*_source_ (case 3, dark-green line). All profiles are normalized by their maximum concentration and shown at 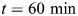.

To test for correlation between asymmetries in the ECS geometry and the Fgf8a gradient, we computed the ECS porosity profiles along the AV and DV axes at 40%, 60% and 75% epiboly. The porosity *ψ* of a porous medium is defined as the ratio between its void volume *V*_void_ and its total volume *V*_total_. We adopt this notion to compute the ECS porosity *ψ*_ECS_ with respect to the embedding spherical shell as
(15)

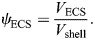
Using Eqn 15, we compute ECS porosity profiles along the AV and DV axis. Figs 12A and 11D show the porosity profiles along the AV and DV axis, respectively, co-plotted with the [Fgf8a-total] profiles for comparison.

Fig. 12A shows that, for all three epiboly stages, the ECS porosity doubles along the AV axis within the first 100 μm from the margin from approximately *ψ*_ECS_≈0.2 to *ψ*_ECS_≈0.4. More than 100 μm from the margin, *ψ*_ECS_ at 40% epiboly increases to approximately 0.5, whereas it decreases to approximately 0.3 at 60% and 75% epiboly. At all stages, the porosity is lowest close to the margin within the band of source cells (70 μm at 40% and 60% epiboly and to 112 μm at 70% epiboly). Lower *ψ*_ECS_ means lower permeability and higher diffusive hindrance. The Fgf8a-gradient kink at the source could therefore be explained by the superposition of the effects of a finite source size (Dalessi et al., 2011) and of higher diffusive hindrance towards the margin.

Along the DV axis, *ψ*_ECS_ and the [Fgf8a-total] profiles are flat at 40% epiboly, as shown in Fig. 12A. The *ψ*_ECS_ DV profile at 60% is similar to the *ψ*_ECS_ DV profile at 75% epiboly, with *ψ*_ECS_ values halving from the ventral to the dorsal side. This gradient anticorrelates with the normalized [Fgf8a-total] DV gradient at 75% epiboly, the concentration value of which doubles between the ventral and the dorsal side, suggesting that the [Fgf8a-total] DV gradient could be explained by the asymmetry of the ECS porosity along the DV axis. This hypothesis, however, is contradicted by the flat [Fgf8a-total] profile at 60% epiboly, despite a similar porosity profile.

In addition to porosity anisotropy, pattern formation in porous media is influenced by the anisotropy of surface reactions (Valdés-Parada et al., 2017). To find out whether the kink in the source region of the Fgf8a profile correlates with source and sink distributions, we aimed to compare the Fgf8a AV profile with a quantity similar to the porosity but for source and sinks along the AV axis. Although, as surface reactions, sources and sinks do not have a volume, we can compute the sum of grid points by which these surfaces are discretized in our image-based model. The volume of this sum of grid points can then be used with Eqn 15 to compute porosity profiles of the source, *ψ*_source_, and the sink, *ψ*_[Fgfr]_, by computing the ratio of their respective volumes and the embedding shell volume.

### 1D model has similar parameter sensitivity but misses kink

To test how Fgf8a gradient formation with the current modeling assumptions differs between the image-based 3D model and a 1D model, we solve Eqns 5-8 in a 1D domain of length *L*=400 μm with the same grid spacing *h*=0.917431 as the 3D model. This domain represents the zebrafish AV axis at 60% epiboly. In contrast to the 3D model, however, it does not resolve cell surfaces and ECS geometry. Instead, we model Fgf8a binding to HSPG^cellSurf^ and Fgfr throughout the 1D domain, and restrict the source to a band of width *w*_source_=70 μm at the margin, according to Eqn 9.

With this setting, and using the same parameters as for the 3D model (Table 2), we simulated *de novo* Fgf8a gradient formation in the 1D domain. The resulting gradient reaches steady state in less than half the time required for the 3D model, i.e. within ≈15 min (see Fig. S9A,B). Fig. 14A shows that, compared with the 3D model, the gradient of the 1D model lacks the kink at the source and is generally flatter. Its decay length *λ*_1D model_=114 μm is more than twice *λ*_in vivo_ and ≈50% larger than 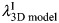, but similar to 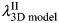.

These differences between the 1D and the 3D model can be explained by the 1D model neglecting the ECS geometry. More specifically, by neglecting diffusive tortuosity, the effective Fgf8a diffusivity in the 1D model is higher than in the 3D model. This faster effective diffusion leads to gradient flattening, as discussed in the section ‘Fgf8a normalized gradients are robust to changes in rate constants but sensitive to changes in ECS geometry’, and to a lower local accumulation time, which has been shown to be approximately proportional to 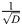 (Gordon et al., 2013). The missing kink close to the margin is likely a consequence of neglecting the spatial heterogeneity and zonation of the ECS geometry near the source.

**Fig. 13.**
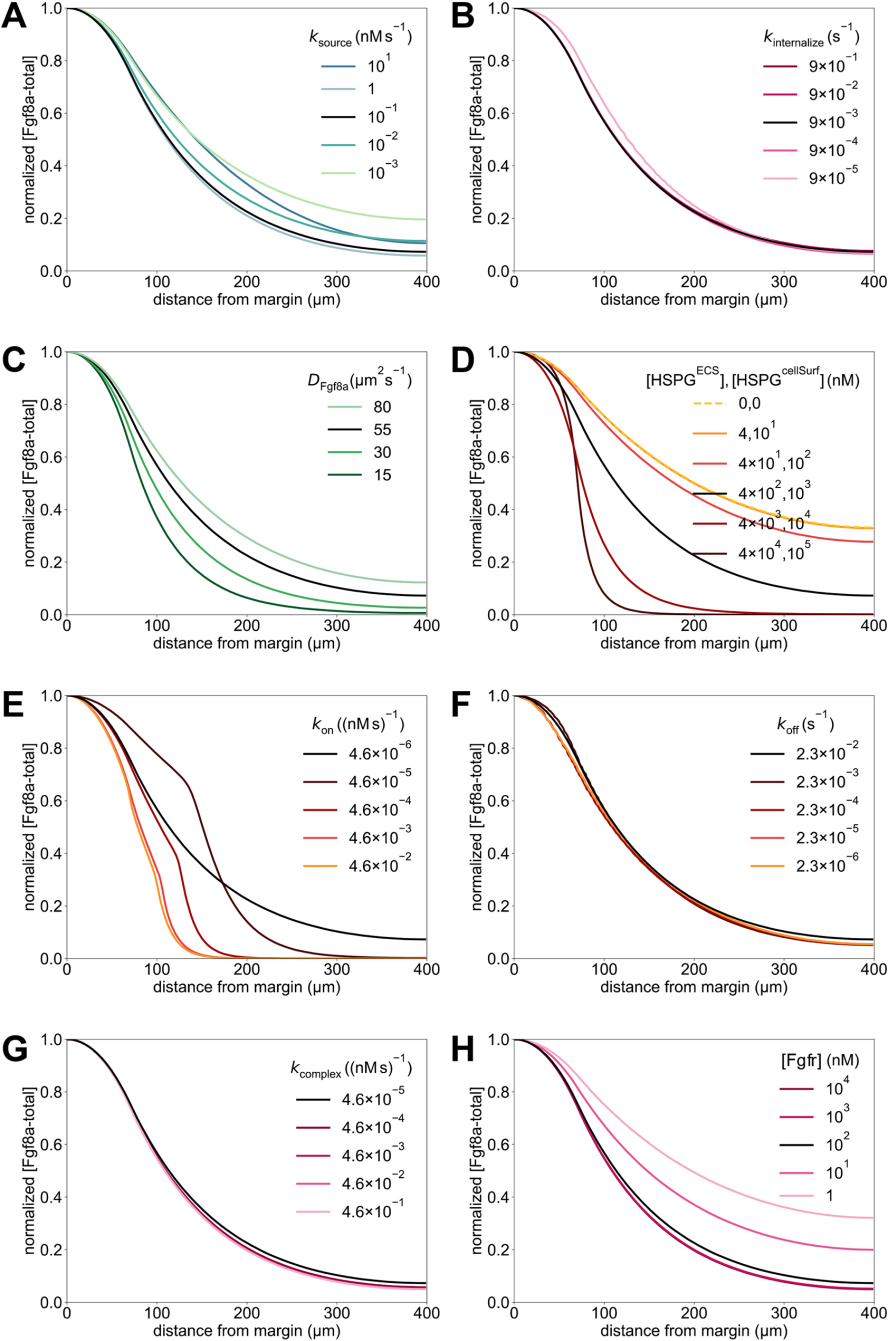
**Sensitivity of a 1D model to variations in the model parameters.** (A,B,G) Similar to the 3D model, the normalized simulated gradient shows robustness against changes in rates of source (A) and sink [i.e. *k*_internalize_ (B) and *k*_complex_ (G)] (see key). (C,D) Changes in the effective diffusivity, by changing either the molecular diffusion coefficient of Fgf8a (C) or HSPG concentrations (D), strongly affect the gradient. The stronger the HSPG binding and the smaller the diffusion coefficient, the steeper and shorter the gradient. (E,F) Higher HSPG affinities (E) lead to shorter and steeper gradients with higher amplitude at the source (see absolute concentrations in Fig. S7E), whereas reducing HSPG unbinding rates (F) does not affect the gradient shape. (H) Compared to the baseline, decreasing [Fgfr] leads to flatter gradients, whereas increasing [Fgfr] has almost no effect on the gradient. This could be because of the HSPG binding that precedes Fgfr binding in our model, which could be the limiting step. For the baseline gradients, shown as a solid black line in all panels, the same parameters were used as in the 3D model (Table 2). All profiles are normalized by their respective maximum concentration. Profiles are shown at 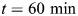, which for complex internalization rates 100 times smaller and all HSPG binding rates larger than the baseline is before the gradient has reached steady state. This agrees with previous analytical solutions showing that steady state is reached slower for higher non-receptor binding (Lander et al., 2007) and smaller degradation rates (Berezhkovskii et al., 2010; Gordon et al., 2013).

To test whether morphogen gradient formation in the porous 3D geometry is more robust against parameter changes than in a 1D domain, we repeated the sensitivity analysis of the section ‘Fgf8a normalized gradients are robust to changes in rate constants but sensitive to changes in ECS geometry’ for the 1D model. Fig. 13 and Fig. S7 show that the normalized Fgf8a gradient in the 1D model reacts similarly to that in the 3D model to most parameter changes. In particular, slower diffusion due to lower Fgf8a diffusivity (Fig. 13C) and higher HSPG binding (Fig. 13D) both lead to steeper and shorter gradients, whereas the normalized gradient is robust against changes in *k*_internalize_ (Fig. 13B) and *k*_complex_ (Fig. 13G). As in the 3D model, the gradient shape robustness against *k*_internalize_ still holds for 10^3^ times larger *k*_complex_, 10^3^ times larger *k*_on_ and 10^3^ smaller *k*_off_, as shown in Fig. S8A,C and E, respectively. Compared to the baseline parameters (Fig. S7B), however, the gradient amplitude is more sensitive to changes in *k*_internalize_ for higher *k*_complex_ and higher *k*_on_, and smaller *k*_off_ (see Fig. S8B,D,F, respectively). This is expected because, in our model, HSPG binding precedes complex formation and internalization. Higher HSPG affinities are therefore expected to increase the sensitivity to internalization rates.

Whereas the gradients of the 1D and 3D model react similarly to changes in the sink rate, the 1D gradient shows higher sensitivity to changes in the source rate (Fig. 13A), with lower *k*_source_ leading to flattening of the normalized gradient. In addition, amplitudes of the absolute 1D gradients change at a four times higher proportionality constant as *k*_source_ changes (see Fig. S4E). This could be explained by the dense source geometry in the 1D model, whereas in the 3D model, the source is fragmented by restriction to cell surfaces, reducing the effective source rate. In addition, fluctuations in the source rate could be buffered by the ECS tortuosity, hindering the diffusion of excess morphogens away from the source and leading to locally higher concentrations. This results in a locally higher probability of Fgfr binding and endocytosis close to the source.

Another difference between the gradients of the 1D and the 3D model is that increasing *k*_on_ leads to a distortion of the gradient shape in the 1D model, with a change of slope appearing between ≈100…200 μm distance from the margin (Fig. 13F). This means that HSPG binding has its strongest impact close to the source, where the morphogen concentrations are highest. That this effect cannot be observed in the 3D model could be explained by the geometric hindrance at the source due to the zonation of the ECS geometry. All profiles in Fig. 13 and Fig. S7 are computed at *t*=60 min, which for complex internalization rates 100 times smaller and for all HSPG binding rates larger than the baseline is before the gradient has reached steady state. This agrees with previous analytical solutions showing that steady state is reached slower for higher non-receptor binding (Lander et al., 2007) and smaller degradation rates (Berezhkovskii et al., 2010; Gordon et al., 2013).

Decreasing [Fgfr] in the 1D model leads to higher absolute concentrations (Fig. S7G), similar to the effect when decreasing *k*_internalize_ and likely a consequence of reduced endocytic degradation. In contrast to the unchanged steepness of the normalized profile for changing *k*_internalize_, however, decreasing [Fgfr] leads to flatter gradients (Fig. 13G). This could be explained by an increase in the Fgf8a effective diffusion coefficient when binding less to Fgfr or by the protection of Fgf8a from molecular degradation by increased complex formation away from the source.

Overall, the gradients of the 1D and the image-based 3D model differ in the kink at the source, which is missing in the 1D model, in the larger decay length in the 1D model, in the higher sensitivity of the 1D model to changes in *k*_source_, and in the gradient distortion in the 1D model for lower *k*_on_. Changes in binding reactions, sink and diffusion parameters have similar effects in the 1D and 3D models.

#### Upscaling source and porosity profiles partially recovers kink in 1D model

To disentangle how the porous geometry is related to the gradient kink at the source, we separately considered different aspects of porous geometries on gradient formation. For this, in addition to the AV porosity profile at 60% epiboly discussed in the section ‘Computing porosity profiles along the AV and DV axes’, we extracted the AV porosity profiles of the source (*ψ*_source_) and sink (*ψ*_[Fgfr]_) from the pore-scale model (see Fig. 12B). We then repeated the 1D simulations for four cases: (1) upscaling (i.e. accounting for the ECS geometry by its AV porosity profile) both *D*_Fgf8a_ and 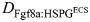 with *ψ*_ECS_, i.e. inhomogeneous diffusion with 
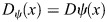
 [following previous works by Wakao and Smith (1962) and Etancelin et al. (2020)]; (2) scaling *f*_source_ with *ψ*_source_; (3) combining (1) and (2); and (4) upscaling *D* with *ψ*_ECS_, *f*_source_ with *ψ*_source_ and [Fgfr] with *ψ*_[Fgfr]_, the last being equivalent to an upscaled sink.

Fig. 12C shows that when only *D* is upscaled (case 1, light-green line) does the gradient become steeper and shorter but maintains its overall shape, i.e. does not develop a kink near the source. When scaling only the source (case 2), the gradient slightly dips close to the margin (light-blue line in Fig. 12C). In case (3), i.e. when upscaling *D* and *f*_source_ by their respective AV porosity profiles, the kink is partly recovered (dark-green line in Fig. 12C). Interestingly, the dip is flatter for case (4), when *f*_source_ also is upscaled in addition to *D* (pink line in Fig. 12C). The reason for this is unclear, but most likely, linear upscaling is not sufficient for representing heterogeneous Fgfr distributions of the real geometry, requiring more sophisticated homogenization approaches (Boso and Battiato, 2013; Dalwadi and King, 2020).

**Fig. 14.**
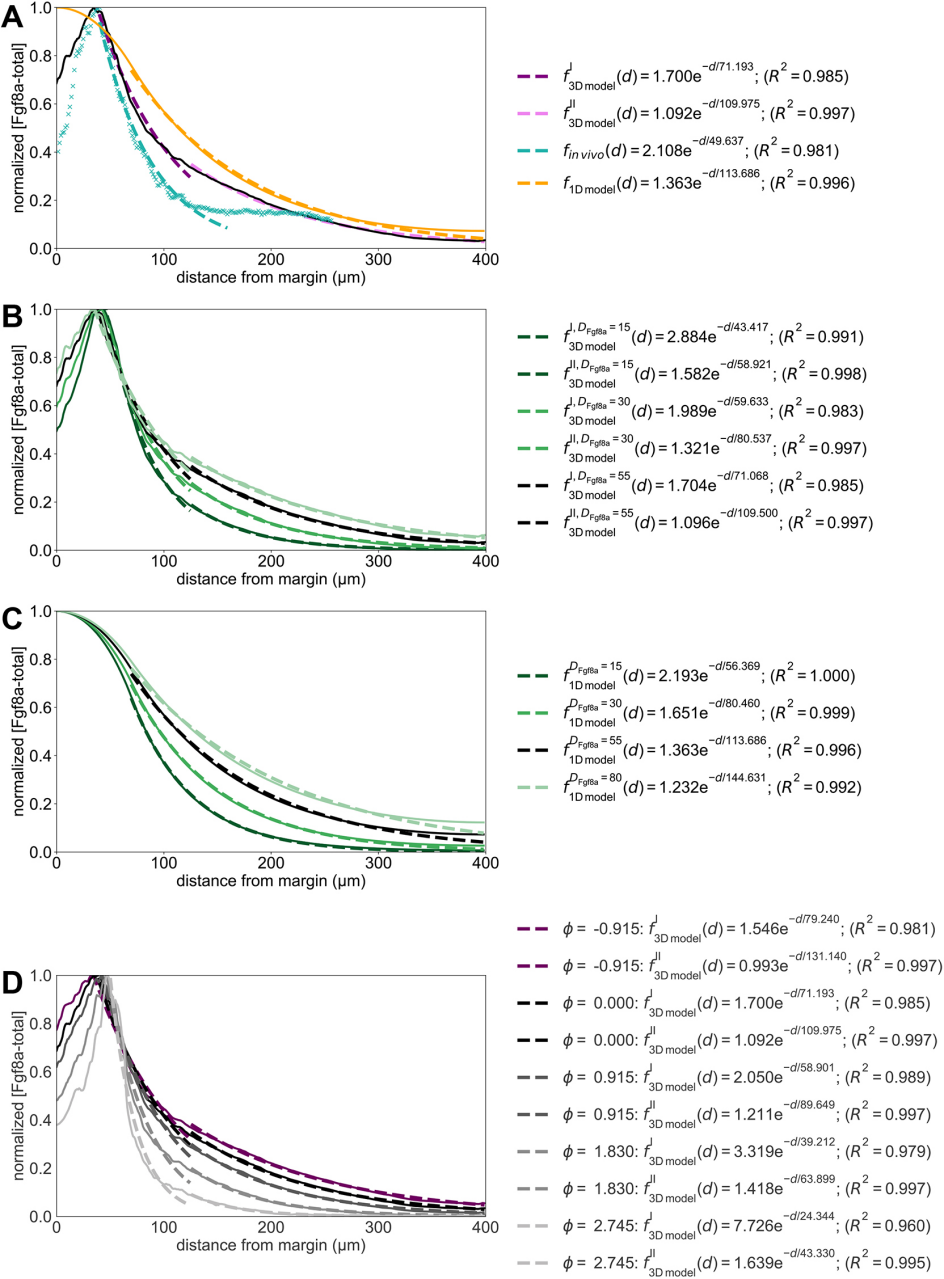
**Fitting exponential decay to *in vivo* and simulated gradients.** (A) Exponential decay fit to *in vivo* 1D and 3D model gradients (dashed lines, see key). The *in vivo* gradient was best fitted in the range 40 μm≤*d*≤161 μm. For the 1D model, the source region was excluded from the fit, i.e. *d*≥70 μm. The gradient of the 3D model was best fitted by a mixture of two exponential functions: the first for 35 μm≤*d*≤125 μm and the second for *d*>125 μm. (B) Fit to image-based 3D simulation gradients for different Fgf8a diffusion coefficients. (C) Exponential decay fit to 1D model simulation gradients for different Fgf8a diffusion coefficients. (D) Exponential fit to gradients of different ECS tube thicknesses. All curves were fitted using the non-linear least squares function curve_fit from the scipy.optimize library (Virtanen et al., 2020). The key provides the fitted proportionality coefficients and their goodness of fit *R*^2^.

### Determining gradient decay lengths

The decay length *λ* of a morphogen gradient was determined by fitting an exponential decay to the gradient
(16)

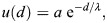
with concentration *u* at distance *d* from the margin and pre-factor *a*. The decay length *λ* of a morphogen gradient is the distance from the location of maximal concentration, 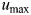, at which *u* has decayed to 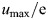 (Kicheva et al., 2007; Grieneisen et al., 2012).

The parameters *a* and *λ* of the exponential decay curves were fitted using the non-linear least squares function curve_fit from the scipy.optimize library (Virtanen et al., 2020). The results are shown in Fig. 14A for the *in vivo* gradient, for the baseline of the image-based 3D model and for the 1D model. In Fig. 14B and C, the fits are repeated for different Fgf8a diffusivities in the 3D and 1D models, respectively. The range over which an exponential function could be fitted differed between the *in vivo* gradient and the two models. The *in vivo* gradient could be fitted by an exponential decay starting from the location of highest concentration at 40 μm from the margin up until about 161 μm from the margin. The fitted decay length was *λ*_in vivo_≈50 μm. The gradient of the 1D model could best be fitted – in line with Dalessi et al. (2011) – when the source region was excluded, i.e. for *d*≥70 μm, resulting in *λ*_1D model_≈117 μm. The gradient of the 3D model could be best fitted by splitting it into two parts: the first starting from the location of highest concentration and extending over 35 μm≤*d*≤125 μm, with 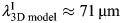, and the second for *d*>125 μm with 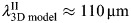. This means that the gradient shape in the first part is closer to the *in vivo* gradient, whereas the second part is more similar to the gradient of the 1D model.

## Supplementary Material

10.1242/develop.204312_sup1 Supplementary information
