# Supplementary material for: Morphogen gradients are regulated by porous media characteristics of the developing tissue
Source: Development. 2025 Jul 14;152(13):dev204312. doi: 10.1242/dev.204312 (PMC12315555; doi:10.1242/dev.204312)
Supplement: Supplementary information [file develop-152-204312-s1.pdf]

S1 Supplementary Methods

S1.1 Grid initialization with experimental gradient

To determine parameters that maintain the *in vivo* gradient, we initialized concentration fields with the experimental intensity profile at 60% epiboly (Fig. 7A)), as this profile has more data points than the FCS count profile shown in Fig. 3E. The intensity profile is one-dimensional and provides average values at discrete locations along the AV axis. In our three-dimensional model, we distribute these values uniformly over xz-planes along the same axis after margin alignment, with a Fgf8a-free / Fgf8a : $\mathrm{HSPG}^{\mathrm{ECS}}$ ratio of 93% : 7%. It has been shown that Fgf8a concentrations are higher at the cell membranes than in the ECS (Gupta et al. 2024); by which factor is, however, unknown, but the factor is not large. To nevertheless represent this difference in our model, we arbitrarily set [Fgf8a : $\mathrm{HSPG}^{\mathrm{cellSurf}}$] to 2× the total ECS Fgf8a concentration. As the spacing between the experimental values does not coincide with the spacing Δy between xz-planes of the image, we linearly interpolate values between neighboring data points. We convert normalized intensity value to concentrations by multiplying with the maximum concentration of 7.9 nM measured by FCS. The resulting concentration field is shown in Fig. 7B).

S2 ECS volume remains almost constant throughout epiboly

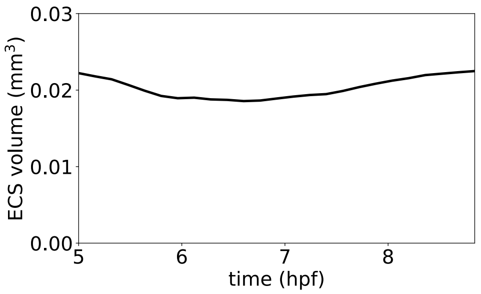

Fig. S1. Reconstructed ECS volume at all 25 time point between 5 hpf (≈40% epiboly) and 9 hpf (≈90% epiboly). The volume was computed by summing the volumes of all grid cells inside the ECS.

It has been proposed that tissue growth contributes to gradient formation by changing the diffusion volume, diluting the morphogen concentration (Wartlick et al. 2009, Aguilar-Hidalgo et al. 2018). We estimate this dilution factor for Fgf8a gradient formation during zebrafish epiboly by computing the total ECS volume for the 3D geometry reconstructions of all 25 time points of the light-sheet microscopy video ranging from 5 hpf (≈30% epiboly) to 9 hpf (≈90% epiboly). The volume is estimated by summing the volumes all grid cells inside the ECS. This volume likely overestimates the real ECS volume, as it is biased by segmentation errors as discussed in Materials and Methods. Nevertheless, as this overestimation error is constant over time, this provides a good estimate of the net change of ECS volume throughout epiboly. We find that the total ECS volume does not increase during the 4 hours of epiboly (Fig. S1), suggesting that the effect of growth-induced morphogen dilution is negligible during epiboly.

Development • Supplementary information

S2.1 The Fgf8a normalized gradient is robust to sink function of source cells

In accordance with *in vivo* observations (Ries et al. 2009, Ota et al. 2010), our model assumes that source cells express Fgfrs, i.e., they simultaneously act as sinks. To test whether the sink function of source cells influences the Fgf8a AV gradient, we simulate *de novo* gradient formation as described in Results but with [Fgfr] = 0 at all source cells. Figure S2A shows that this increases the absolute peak concentration. This is expected, as less morphogen is degraded in the source region. After normalization, however, the source-is-sink and source-not-sink profiles almost coincide, with a slightly shorter range in the source-not-sink gradient. This shows that the impact of this assumption on the overall simulation results is negligible.

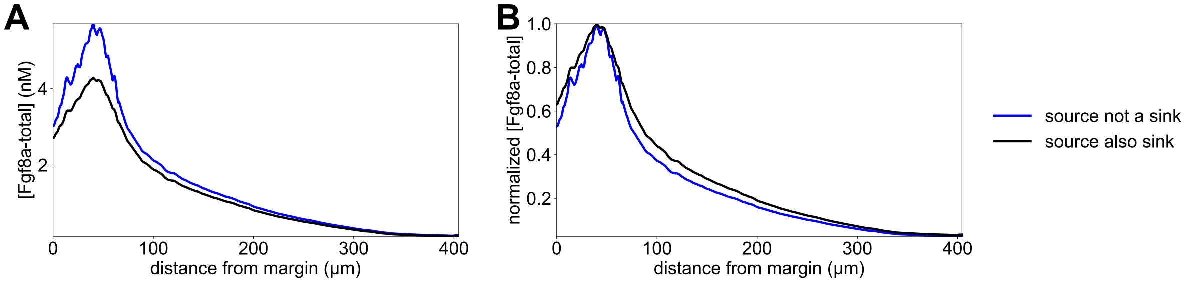

Fig. S2. Simulated AV concentration profiles of *de novo* Fgf8a gradient formation when source cells are not sink cells (blue, inset legend) compared to the baseline assumption that sources simultaneously act as sinks (black line). Concentration profiles before (A) and after (B) normalization are shown at $t = 60$ min.

S3 Supplementary figures

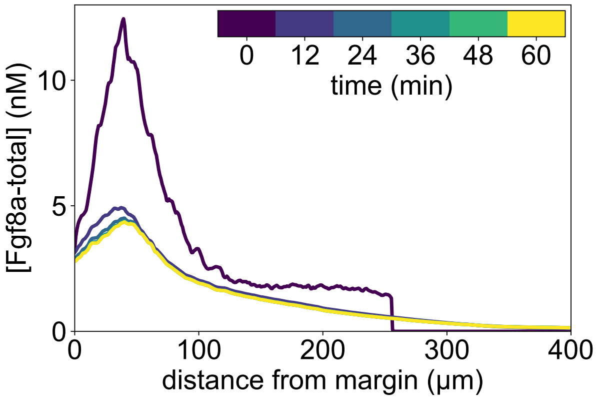

Fig. S3. Simulated Fgf8a gradient using the optimized pa-rameters from Table 2 with experimental profile as initial condition. Absolute AV concentration profiles of Fgf8a-total at different simulated times (color). The experimental profile used as initial condition is shown in black (time 0).

Development • Supplementary information

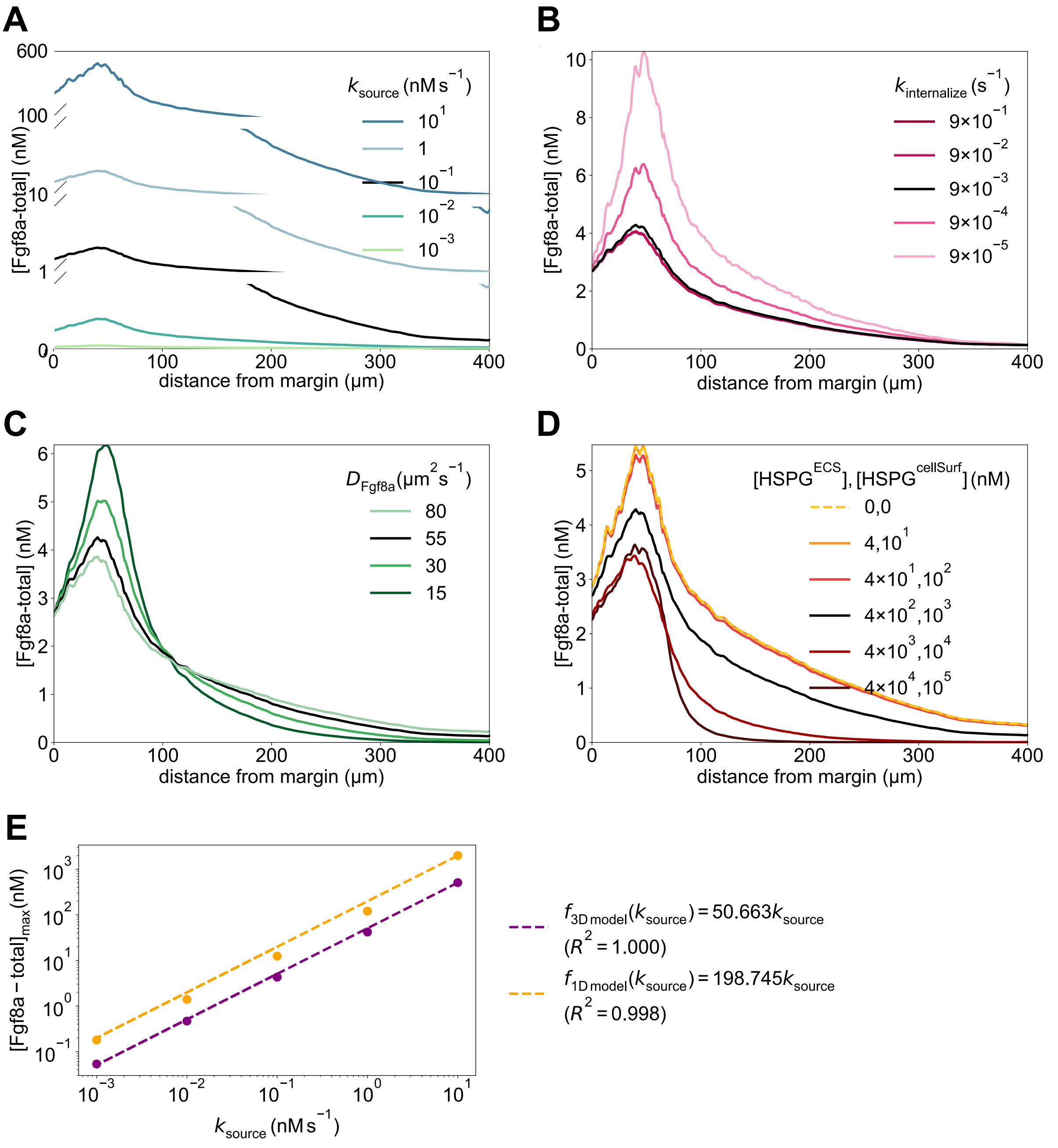

Fig. S4. Absolute concentration profiles for the robustness tests of simulated *de novo* Fgf8a gradient formation. The corresponding normalized profiles are shown in Fig. 3. Peak concentrations are higher for larger source rates (A) and for lower sink rates (B) (color, inset legend). Peak concentrations are proportional to the source rate in the 3D and 1D models (E, inset legend). Changes in the effective Fgf8a diffusivity, by changing either the molecular diffusion coefficient of Fgf8a (C) or the HSPG concentrations (D), strongly affect the gradient shape in addition to the peak concentration. The more HSPG binding and the smaller the diffusion coefficient, the steeper and shorter the gradient. The baseline gradient for the nominal parameters from Table 2 is shown as a solid black line in all panels. The y-axis of (A) is broken into segments of different scales to enable visualizing concentration values across three orders of magnitudes. All profiles are shown at $t = 60$ min.

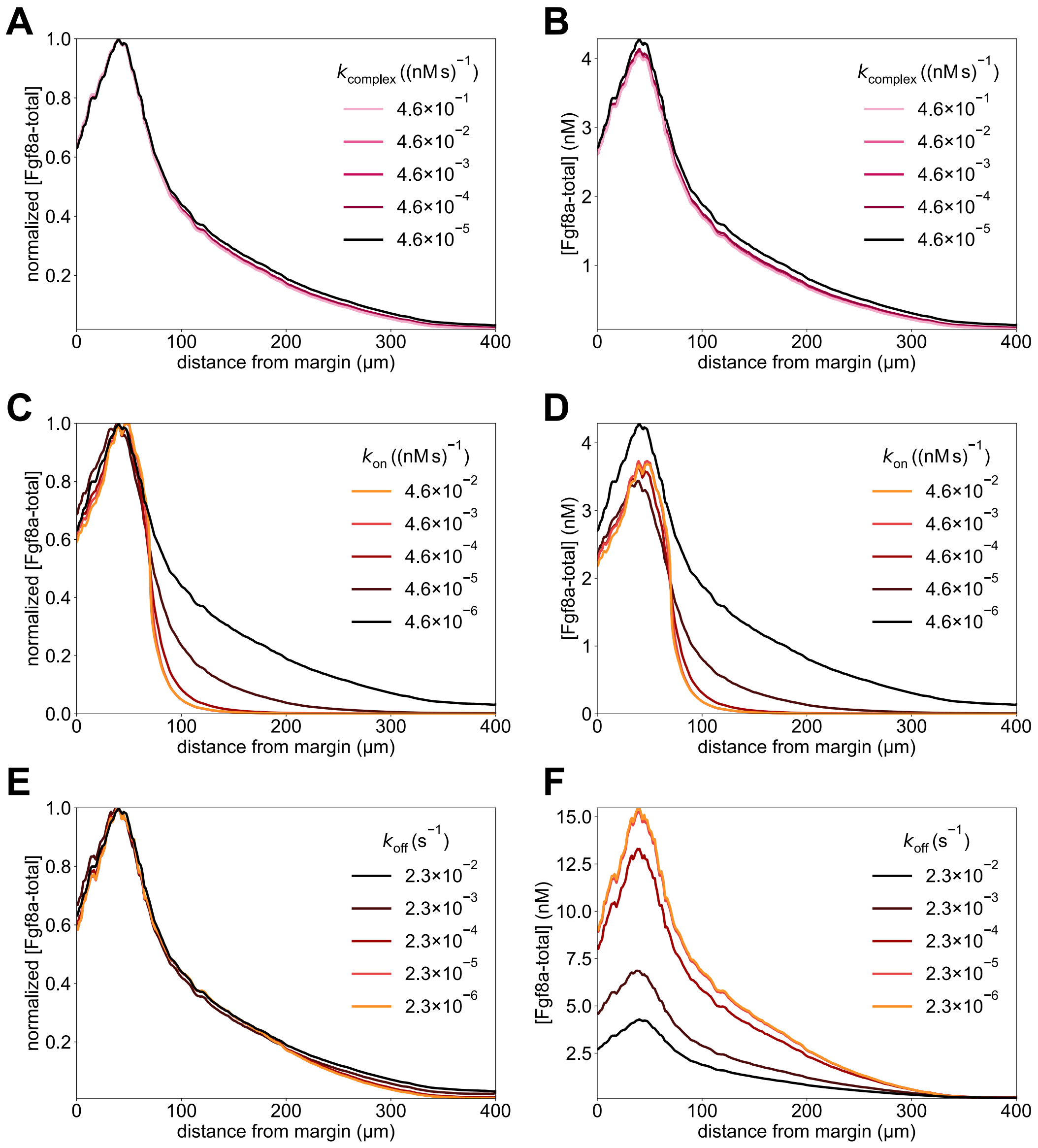

Fig. S5. Binding rates sensitivity analysis. Normalized (A, C, E) and absolute (B, D, F) concentration profiles of the image-based 3D model for different values of k_{complex} (A, B), k_{on} (C, D), and k_{off} (E, F) (color, inset legend), keeping the other parameters fixed as in Table 2. The normalized and the absolute profiles are robust against changes in k_{complex}. A possible explanation for this is that complex formation protects Fgf8a from proteolysis in the ECS, thereby counterbalancing molecular decay. Increasing k_{on} leads to shorter and steeper gradients with concentration levels that are highest for the baseline value, lowest for 10 times larger k_{on}, and in between for $10^2 - 10^4$ times larger k_{on}. Similar to k_{complex}, this could be explained by HSPG binding protecting Fgf8a from proteolysis. Decreasing k_{off} leads to higher gradient amplitudes, as is expected for reduced HSPG affinities, whereas the normalized profile is not affected by reducing k_{off}. All profiles are shown at $t = 60\,\mathrm{min}$.

Development • Supplementary information

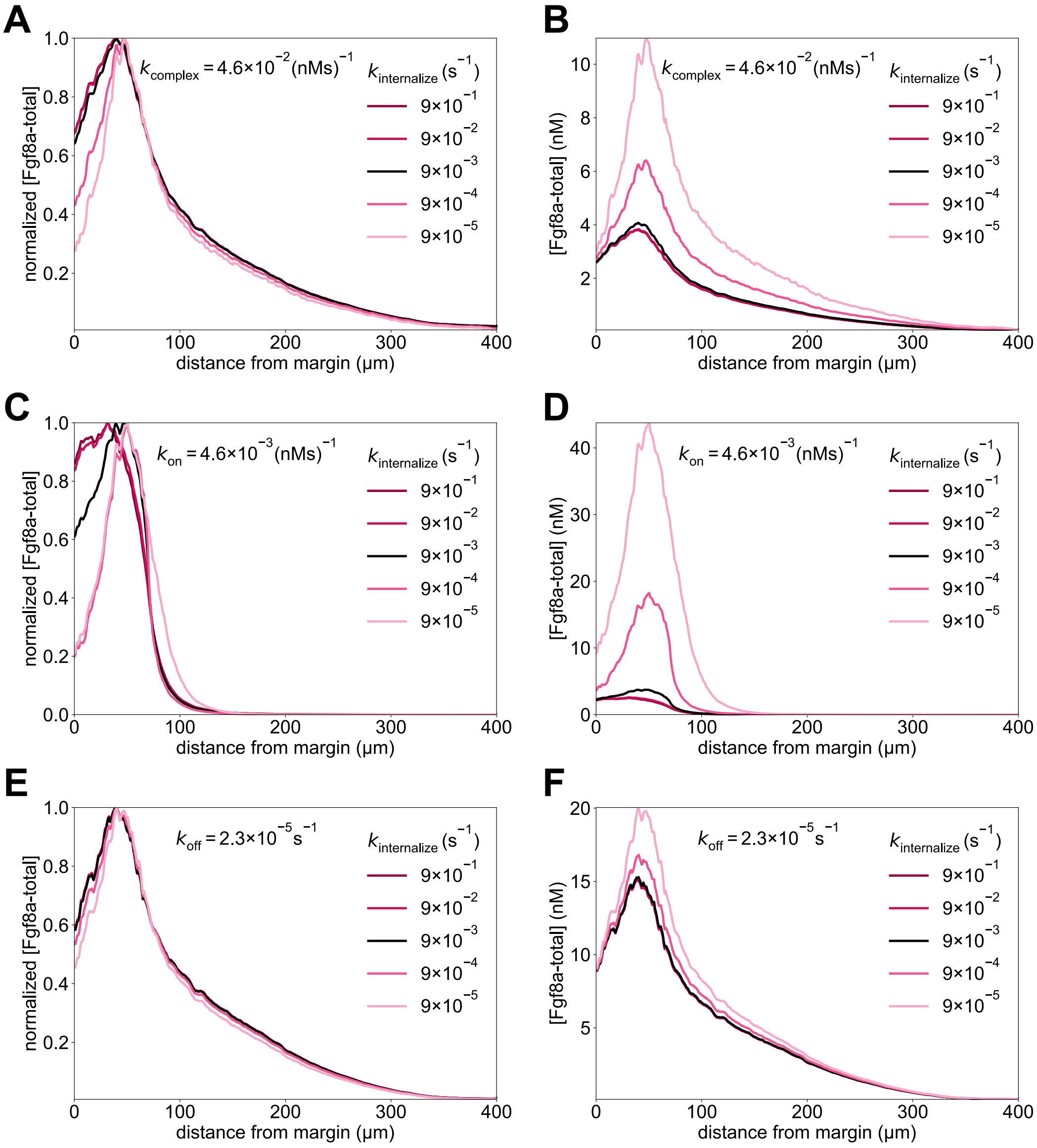

Fig. S6. Sink additional sensitivity analysis for the 3D model. Normalized (A,C,E) and absolute (B,D, F) concentration profiles of the image-based 3D model for di!erent values of $k_{internalize}$ for 103 times larger $k_{complex}$ (A, B), 103 times larger kon, and 10^3 times smaller ko! (E, F) keeping the remaining parameters fixed as in Table 2. In all cases, the normalized gradient is robust against changes in kinternalize (A, C, E). Compared to the sink robustness test with baseline parameters (Fig. S4B), however, the gradient amplitude is more sensitive to changes in $k_{internalize}$ for larger $k_{complex}$, larger kon, and smaller k_{off} (B, D, F). This is expected since, in our model, HSPG binding precedes complex formation and internalization. Therefore, higher HSPG a"nities are expected to increase the sensitivity to internalization rates. All profiles are shown at t = 60min.

Development • Supplementary information

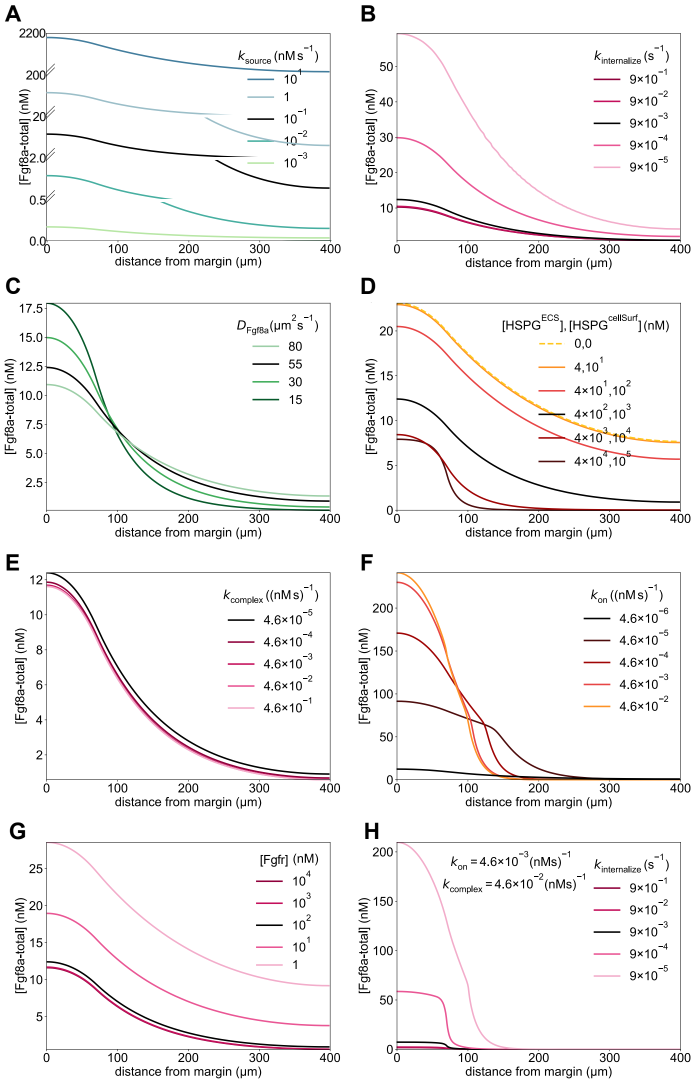

Fig. S7. Absolute concentration profiles for the 1D model sensitivity analysis. Similar to the 3D model, the amplitude of the simulated gradient increases when increasing the source (A) and decreasing the complex internalization (B) rates (color, inset legends). The relative change in amplitude is, however, almost four times larger than in the 3D model (see Fig. S4E) Smaller molecular diffusion coefficients of Fgf8a (C) lead to higher amplitudes at the source and steeper gradients. Higher HSPG (D) and Fgfr (H) concentrations lead to higher concentration levels even at larger distances from the source. Increasing the HSPGbinding rates leads to higher amplitudes and steeper gradients, whereasreducingHSPGunbinding rates (F) increases the amplitudes without affecting the gradient shape (see normalized gradients in Fig. 13F). Increasing the complex formation rate (G) has almost no effect on the gradient. For the baseline gradients, shown as a solid black line in all panels, the same parameters were used as in the 3D model (Table 2). The y-axis of (A) is broken into segments of different scales to enable visualizing concentration values across four orders of magnitudes. Profiles are shown at $t = 60$min, which for complex internalization rates 100 times smaller and all HSPG binding rates larger than the baseline is before the gradient has reached steady state. This agrees with previous analytical solutions showing that steady state is reached slower for higher non-receptor binding (Lander et al. 2007) and smaller degradation rates (Berezhkovskii et al. 2010, Gordon et al. 2013).

Development • Supplementary information

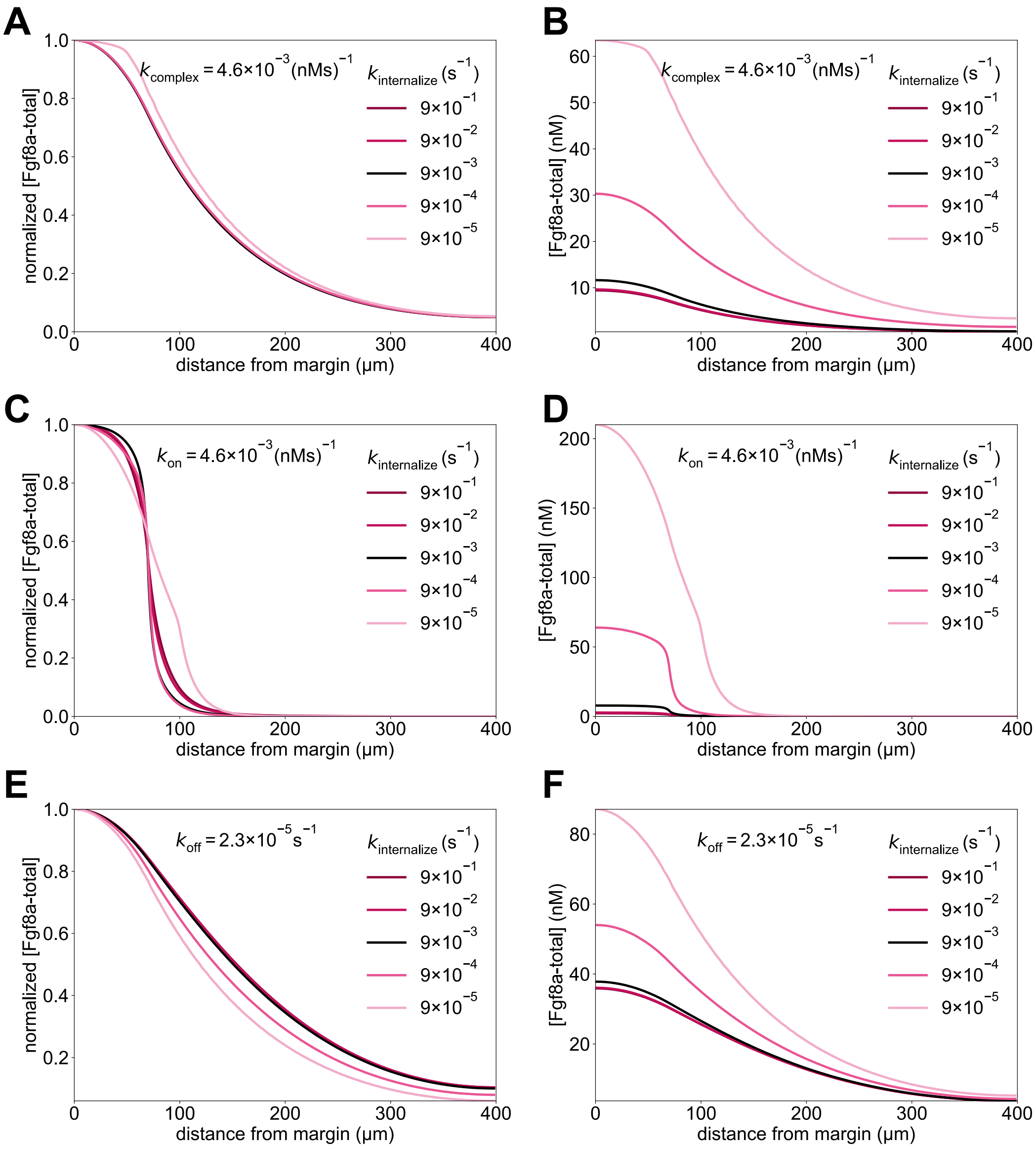

Fig. S8. Sink additional sensitivity analysis for the 1D model. Normalized (A,C,E) and absolute (B,D,F) concentration profiles of the 1D model for different values of $k_{\text{internalize}}$ for 10^3 times larger k_{complex} (A, B), 10^3 times larger k_{on} (C, D), and 10^3 smaller k_{off} (E, F) keeping the remaining parameters fixed as in Table 2. Similar to the 1D sink robustness test using the baseline parameters shown in Fig. S7B, the normalized gradient is robust against changes in $k_{\text{internalize}}$ (A, C, E). Similar to the 3D model (Fig. S6), the 1D gradient amplitude is more sensitive to changes in $k_{\text{internalize}}$ for higher rates of receptor complex formation (B), HSPG binding (D), and lower HSPG unbinding rates (F) in comparison with using the baseline parameters (Fig. S7B). This is expected since, in our model, HSPG binding precedes complex formation and internalization. Therefore, higher HSPG affinities are expected to increase the sensitivity to internalization rates. All profiles are shown at $t = 60\ min$.

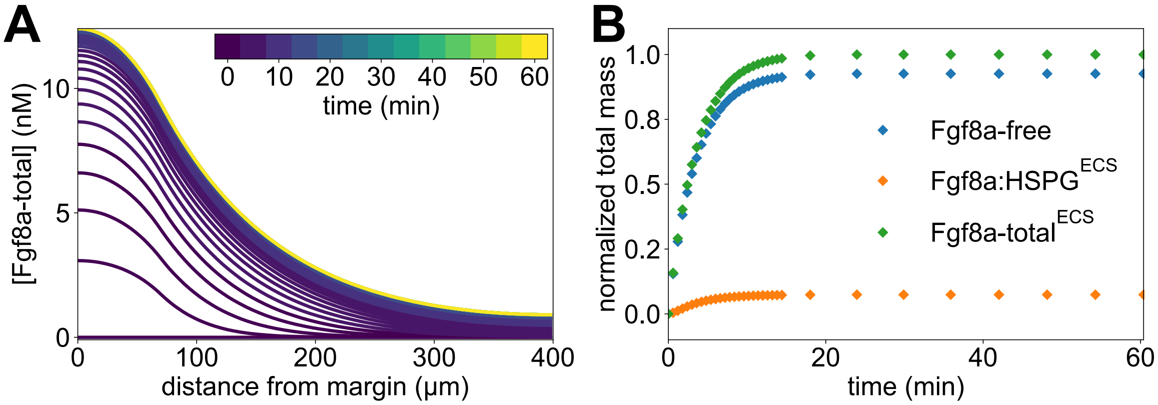

Fig. S9. Simulated Fgf8a gradient formation of 1D model. (A) AV concentration profiles of Fgf8a-total at different simulated times (color bar) using the baseline parameters found for the 3D model (Table 2) and model assumptions described in Materials and Methods. Simulated profiles reach a steady state within 15 min. (B) Normalized total mass of two Fgf8a fractions $\mathrm{Fgf8a : HSPG^{ECS}}$ and Fgf8a-free, along with their sum $(\mathrm{Fgf8a - total^{ECS}})$.

S4 ECS reconstructions at all 25 time points

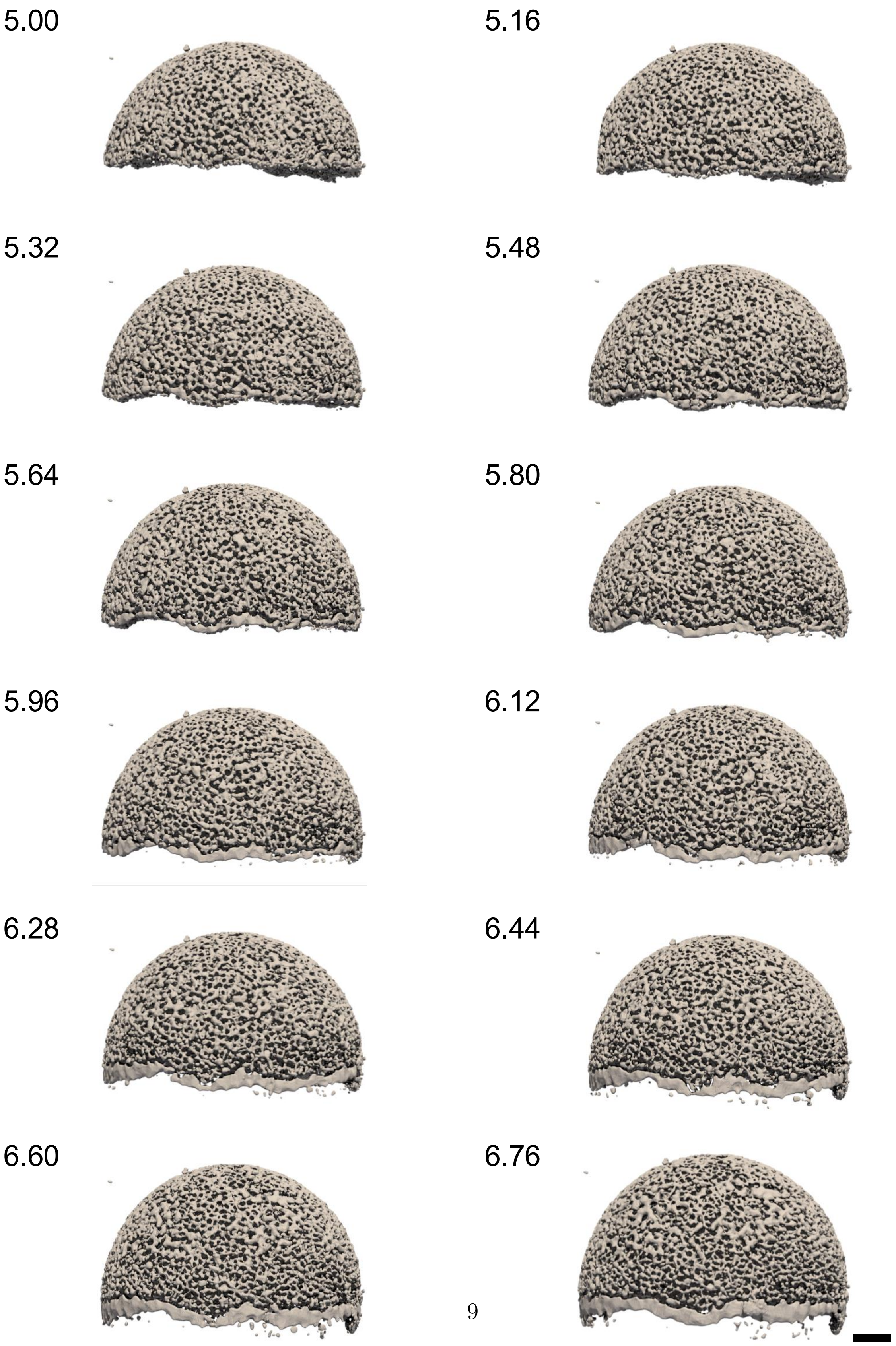

Development • Supplementary information

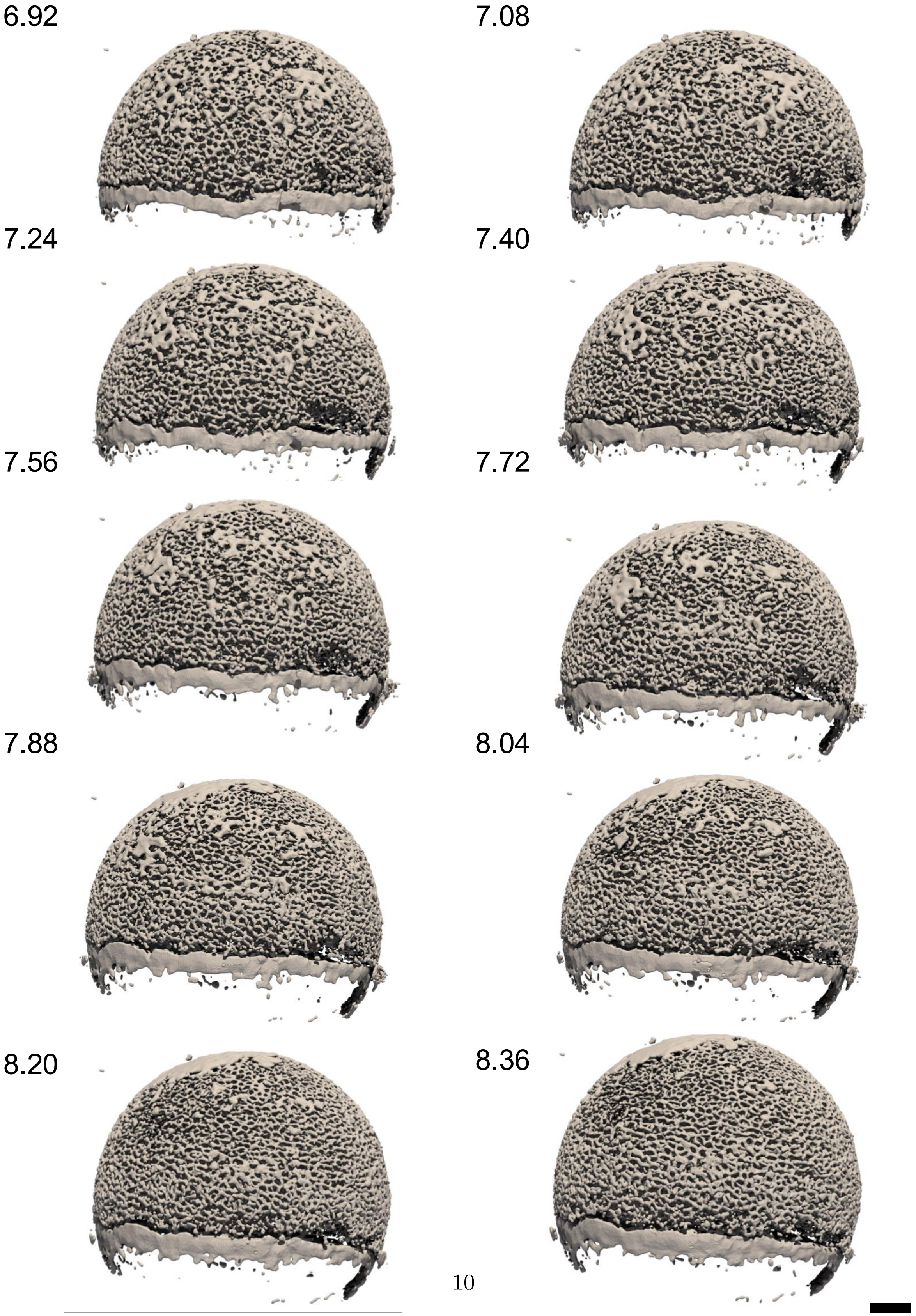

Development • Supplementary information

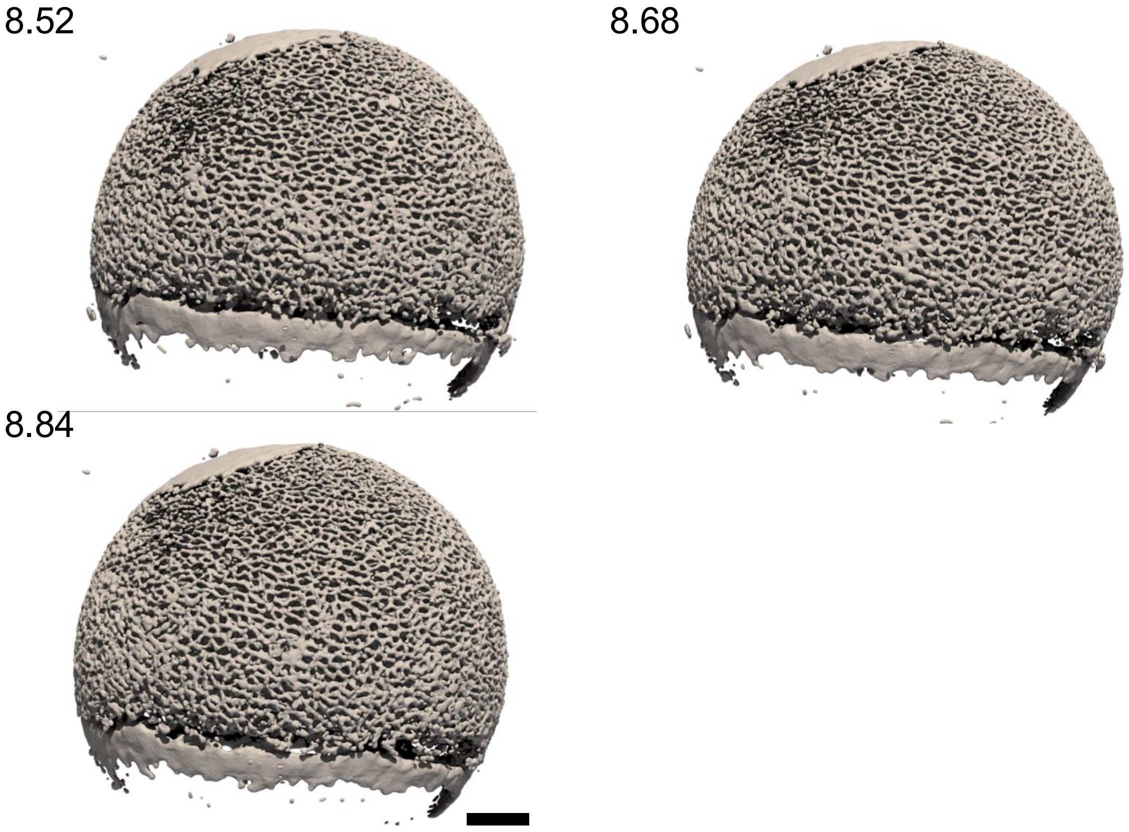

Fig. S10. Visualization of the signed-distance-function representation of the ECS boundary at all 25 time points of the light-sheet microscopy video, ranging from 5 hpf (≈30% epiboly) to 9 hpf (≈90% epiboly). For each time point, we show a surface rendering of $\phi_{\mathrm{ECS}} = 0$ in gray after margin alignment. Numbers are the approximate hours post-fertilization (hpf) at the start of each respective frame acquisition. All scale bars: 100 µm.

Development • Supplementary information

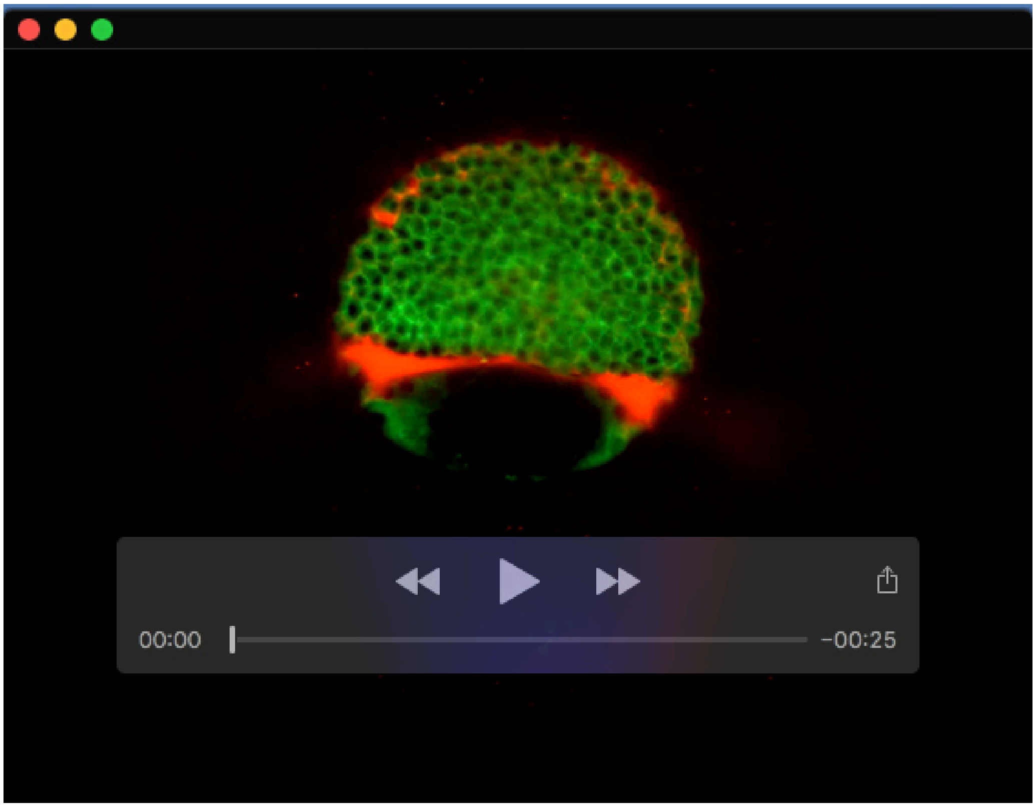

Movie 1. Light-sheet microscopy time-lapse video of zebrafish epiboly. Exemplary optical sections from a Tg(bactin:hRas-EGFP) embryo at 25 time points acquired from 5 hpf (≈40% epiboly) to 9 hpf (≈90% epiboly), respectively, following image acquisition using a light-sheet fluorescence microscope and multi-view reconstruction described in Section 4.4. hRas-EGFP labels the cell membranes (green). ECS is marked by TMR-Dextran injection (orange).
